# Internalization Characterization of Si Nanorod with Camouflaged Cell Membrane Proteins Reveals *ATXN2* as a Negative Regulator

**DOI:** 10.3390/cells8080931

**Published:** 2019-08-19

**Authors:** Yi Lu, Jing Dai, Na Kong, Jianghuai Liu, Jinkang Gong, Yuan Yao

**Affiliations:** 1 State Key Laboratory of Pharmaceutical Biotechnology and Ministry of Education Key Laboratory of Model Animals for Disease Study, Model Animal Research Center of Nanjing University, Nanjing 210061, China; 2 School of Physical Science and Technology, ShanghaiTech University, 393 Middle Huaxia Road, Shanghai 201210, China; 3 Shanghai Institute of Ceramics, Chinese Academy of Sciences, Shanghai 200050, China; 4 University of Chinese Academy of Sciences, Beijing 100049, China

**Keywords:** silicon nanorod, cell membrane proteins, cancer, internalization, efficiency, *ATXN2*

## Abstract

The fabrication of shape-controlled nanocarriers is critical for efficient delivery of biomolecules across the cell membrane. Surface coating of the nanocarrier can improve internalization efficiency. Here, we developed a facile method of silicon nanorod fabrication leading to a controlled size and shape. We then systematically evaluated five surface modifications with membrane proteins from different cancer cell lines including MCF7, MD231, Hela, Panc-PDX, and Panc-1. We demonstrated that silicon nanorods coated with either a homolytic or heterolytic membrane protein coating have significantly improved internalization efficiency as compared with uncoated Si nanorods. To elucidate the molecular mechanism of the improved efficiency associated with a modified coating, we analyzed the coating membrane proteins derived from five cell lines with proteomics and identified 601 proteins shared by different cell sources. These proteins may function as cell-substrate adhesion molecules that contribute to the enhanced internalization. We also tested the internalization efficiency of nanorods with different coatings in each of the five cell lines to determine the influencing factors from target cells. We found that the internalization efficiency varied among different target cells, and the ranking of the average efficiency was as follows: Hela > Panc-PDX > MD231 > MCF7 > Panc-1. The bioinformatics analysis suggested that the low internalization efficiency in Panc-1 cells might be associated with the upregulation of *ATXN2*, which is a negative regulator of endocytosis. We further demonstrated that *ATXN2* knockdown with specific siRNA significantly improved nanorod internalization efficiency in Panc-1 cells suggesting that *ATXN2* can be a reference for efficiency prediction of nanoparticle delivery to tumor cells. Thus, we studied the effect of different cancer cell membrane proteins on nanorod uptake efficiencies. These results can improve nanorod internalization to cancer cells, including a fundamental understanding of the internalization efficiency of cancer cells.

## 1. Introduction

Nanoparticles have been used therapeutically in various diseases [1,2]. The interaction between nanoparticles and target cells is critical to the understanding of the internalization efficiency in cells and tissues. The physicochemical properties of nanoparticles, such as shape, size, and surface modification, often determine the internalization dynamics [3,4]. For example, particle shape and size can modulate the internalization efficiency of nanoparticles [5]. Surface modification can also promote the nanocarrier properties after functionalization with chemical moieties, antibodies, or membrane proteins, i.e., active targeting [6,7].

Nanoengineering of medical carriers has been developed various frameworks [2,8,9,10,11]. Many studies have reported wide-ranging internalization efficiencies of different nanocarriers, which warrant further investigation. Silicon nanostructures have broad potential applications in the biomedical and clinical industries because of their good biocompatibility [12], strong semi-conductive properties, and easy fabrication ability [13]. Furthermore, the rod shape is associated with higher uptake efficiency than that of spherical, plate, or flake-like structures [11,14].

Recently, several studies have used biomimetic nanoparticles with a plasma membrane as a coating material to improve uptake efficiency [6,7,15,16,17,18,19,20,21,22,23,24]. The sources of the cell membrane include platelets [20,25,26,27], red blood cells [28], white blood cells [29,30,31], stem cells [32,33], and cancer cells [16,17,34,35]. It has been reported that cancer cell membrane coating nanoparticles may have better internalization efficiency in targeted cancer therapy [16,36].The applications have also been expanded to immune privilege, antigen presentation, drug uptake, detoxification, imaging, photoactivatable therapy, self-homing, and tumor targeting therapies [16,17,22,33,35,37,38]. Despite this progress, the factors affecting the internalization efficiency of nanoparticles coated with different membrane proteins remain obscure. It is critical to identify and quantify these factors with molecular mechanistic insight.

Here, we developed a facile method for the nanofabrication of Si nanorods (SiNR) with precisely controlled sizes to investigate the internalization efficiency of SiNRs with different coatings derived from membrane proteins of five cancer cell lines (Hela, Panc-PDX, MD231, MCF7, and Panc-1). We found that both homolytic and heterolytic membrane coatings significantly enhanced internalization. Testing the effect of different target cells on the internalization efficiency of coated SiNRs revealed that the efficiency varied significantly among different cell lines wth Hela being the highest and Panc-1 the lowest. We then used bioinformatics and siRNA approaches to demonstrate that the upregulation of Ataxin 2 (*ATXN2*), a negative regulator of endocytosis, is a critical factor associated with the low SiNR internalization efficiency in Panc-1 cells.

## 2. Materials and Methods

### 2.1. Fabrication of Nanorods

The silicon nanorod arrays were fabricated via a metal-assisted chemical etching method [39]. A P-type (100) oriented silicon wafer (electrical resistivity, 10–20 Ω·cm) was used in this research. The wafers were first ultrasonically cleaned in acetone and ethanol to remove surface contamination. The wafers were then immersed in boiling piranha solution (3:1, *v/v*, H_2_SO_4_/H_2_O_2_) for 1 h to remove all organic contamination followed by a thorough wash with ultrapure water. Subsequently, monolayer polystyrene (PS) spheres (500 nm diameter) were coated on the silicon wafer via a self-assembly procedure. The PS spheres were then decreased to 400 nm dimeter via oxygen plasma etching (5 sccm oxygen, 100 W power) for 10 min. Thereafter, a 5 nm titanium layer was electron beam evaporated on the PS monolayer followed by 30 nm of silver thermal evaporated on the titanium layer. The PS spheres were removed by ultrasonic cleaning in ultrapure water for 1 min, and then the wafer was immersed in an etching solution (4.6 M HF and 0.44 M H_2_O_2_ solution) for 10 min to get nanorods array. Silver was removed by HNO_3_ for 1 h, and the organic residual was removed by chloroform for 1 h. Finally, the nanorods were scraped off the silicon wafer via a surgical blade and dispersed in ultrapure water for use.

Some nanorods were further amine coated for membrane coating. Here, the wafer was first oxidized by boiling piranha solution (3:1, *v/v*, H_2_SO_4_/H_2_O_2_) for 90 min. Then, nanorods were stepwise cleaned with DI water, methanol, methanol/dichloromethane (DCM) (3:1, *v/v*), and dichloromethane. Subsequently, nanorods were immersed in (3-aminopropyl) triethoxysilane (APTES, 20% in dichloromethane, *v/v*) and reacted for 12 h in nitrogen atmosphere. The nanorods were then sequentially cleaned with ethanol, isopropanol, and DI water and then nitrogen flow dried. The amino-modified silicon nanorods (SiNR-NH2) can be stored dry at 4 °C for at least one month.

### 2.2. Characterization of SiNRs by SEM and TEM

The Si nanorod arrays were observed using scanning electron microscopy (SEM) (JSM-7800F, JEOL, Tokyo, Japan). The coated proteins of SiNRs were examined with transmission electron microscopy (TEM) (JEM-1400plus, JEOL, Tokyo, Japan). Briefly, SNR aliquots (2.5 µL) were transferred onto 200 mesh Cu grids, and the excess moisture was removed with filter paper after 3 min of deposition. The grid was subsequently soaked in 1% uranyl acetate solution (Beijing Zhongjingkeyi Technology Co., Ltd., Beijing, China) for one minute, and the solution was then removed with filter paper. Finally, the grid was dried in a vacuum overnight for TEM characterization. The absorption spectra were characterized with UV-vis spectrometry (U3010, TECHCOMP LTD, Tokyo, Japan). The hydrodynamic size was measured on a nanosizer (NanoZS, Malvern, UK).

### 2.3. Cell Culture

The Panc-PDX cells were primary cells from a pancreatic cancer patient-derived xenograft mouse model (a gift from Dr. Wentao Dai in Shanghai Center for Bioinformation Technology, Shanghai Industrial Technology Institute). Panc-1, MCF-7, MDA-MB-231, and Hela were established cancer cell lines obtained from the cell bank of the Chinese Academy of Sciences (Shanghai, China). All five cell lines were maintained in Dulbecco’s modified Eagle medium (DMEM) with 10% fetal bovine serum (FBS) without antibiotics.

### 2.4. Cell Membrane Protein Extraction

For the cell membrane protein extraction, all cell types were seeded in 15 cm plates. When they reached confluence, cells were harvested using a scraper in cold PBS and isolated by centrifugation at 700 *g* for 5 min. The supernatant was aspirated, and the cell pellets were frozen at −80 °C. The ProteoExtract^®^ Native Membrane Protein Extraction Kit (Millipore, USA) was employed following the manufacturer’s instructions to obtain nondenatured functional membrane proteins. In brief, the cell pellet was washed twice with the washing buffer, and then incubated with ice-cold Extract Buffer I at 4 °C for 10 min under gentle agitation. The pellet was centrifuged at 16,000 *g* for 15 min (4 °C). The supernatant was discarded, and 1 mL ice-cold Extract Buffer II was added to the pellet. This membrane protein extraction step was allowed for 30 min at 4 °C under gentle agitation. Then the supernatant was collected after centrifugation at 16,000 *g* for 15 min at 4 °C. The membrane extracts were characterized with a BCA Protein Assay kit (Takara, Dalian, China) and then stored at −80 °C for SiNR coating.

### 2.5. SDS-PAGE and Silver Staining

For each sample, 20 µL protein extractions were loaded on 4%–20% ExpressPlus™ PAGE Gel (Genscript, Nanjing, China) followed by electrophoresis. Silver staining used a rapid silver staining kit (Beyotimes, Shanghai, China) for detection.

### 2.6. Western Blot

The concentrations of protein extractions were determined using a BCA Protein Assay kit (Takara, Dalian, China). Here, 10 μg protein extractions were loaded onto each lane of a denaturing 4%–20% gradient gel and fractionated. Proteins were transferred to the Hydrophobic Polyvinylidene Fluoride (PVDF) membrane, and the blot was probed with an N-cadherin (610920, BD Pharmingen, San Diego, CA, USA), E-cadherin (610181, BD Pharmingen, San Diego, CA, USA), vimentin (5741S, Cell signaling, Danvers, MA, USA), GAPDH (60004-1-Ig, Proteintech, Wuhan, China), plectin (ab32528, Abcam, Cambridge, England), CDC42 (2462S, Cell signaling, Danvers, MA, USA), pan-keratin (4545S, Cell signaling, Danvers, MA, USA), β-catenin (610153, BD Pharmingen, USA), β-Actin (A00702-100, Genscript, Nanjing, China) and antibody, *ATXN2* (21776-1-AP, Proteintech, Wuhan, China). Western blots were imaged and quantitated with a Bio-Rad ChemiDoc XRS+ System.

### 2.7. LC-MS/MS

The proteins were precipitated with trichloroacetic acid solution (TCA, 6.1 N). The pellet was subsequently dissolved in 8 M urea and 100 mM Tris-HCl, pH 8.5. TCEP (final concentration of 5 mM, Thermo Scientific, Waltham, MA, USA) and iodoacetamide (final concentration of 10 mM, Sigma-Aldrich, St. Louis, MO, USA) were added to the solution and incubated at room temperature for 20 min and 15 min for reduction and alkylation, respectively. The protein mixture was diluted by a factor of four and digested with Trypsin at 1:50 (*w/w*) (Promega, Madison, WI, USA). The reaction was stopped by formic acid (FA), and the peptide mixture was desalted by monospin C18 column (SHIMADZU-GL, Tokyo, Japan).

The peptide mixture was loaded onto a homemade 15 cm long pulled-tip analytical column (aqua, C18, 750 μm OD × 360 μm ID, 3μm particle size, 125Å pore diameter, Phenomenex, Torrance, CA) connected to an Easy-nLC 1000 nano HPLC (Thermo Scientific, San Jose, CA, USA) for mass spectrometry analysis. The mobile phase and elution gradient used for peptide separation were as follows: 0–5 min, 0%–2% B; 5–170 min, 2%–35% B; 170–180 min, 35%–80% B; 180–190 min, 80% B, 190–191 min, 100%–0% B; 191–200 min, 0% B (buffer A: 0.1% FA in water and buffer B: 0.1% FA in acetonitrile) at a flow rate of 300 nL/min. Peptides eluted from the LC column were directly electrosprayed into the mass spectrometer with a distal 1.8 kV spray voltage. Survey full scan MS spectra (from 300–1800 *m/z*) were acquired in the Orbitrap analyzer with resolution r = 70,000 at *m/z* 400. The top 20 MS/MS events were sequentially generated and selected from the full MS spectrum at a 30% normalized collision energy.

The acquired MS/MS data were analyzed against UniProt *Homo sapiens* database (http://www.uniprot.org/) by Sequest algorithm integrated in the Protein Discoverer software (Thermo Scientific). A decoy database containing the reversed sequences of all the proteins was appended to the target database to accurately estimate peptide probabilities and false discovery rate (FDR); the FDR was set at 0.01.

### 2.8. GO Enrichment Analysis

The proteins identified from the MS data were further filtered as the parameter ‘unipeptide’ greater than or equal to 1. The Venn plot analysis were calculated by jVenn online tool [110]. The bioinformatics processes were carried out using R and Bioconductor tools. Briefly, the ID mappings were based on R package “org.Hs.eg.db” and the Gene ontology (GO) cluster and enrichment analysis were performed by R package “clusterProfiler” [111].

### 2.9. Membrane Protein Coating and Labeling

To fabricate membrane protein coated silicon nanorods, SiNR-NH_2_ was firstly reacted with thiophosgene in (1% in acetonitrile (MeCN), *v/v*) for 2 h at room temperature. The phosgene-modified silicon nanorods, SiNR-NCS, can be stored dry at 4 °C for several months. After phosgene modification, the nanorods were immersed in a membrane protein solution (0.2 mg/mL in PBS) for 12 h at 4 °C on a shaker. Protein-modified nanorods were labeled with 5-carboxyfluorescein N-succinimidyl ester (5-FAM-SE, 1 mg/mL in 0.1 M NaHCO_3_ solution, ex/em = 494/522 nm) for 2 h at room temperature. Finally, the membrane protein-coated SiNRs were washed with PBS at least 3 times. Fluorescence-labeled SiNRs without membrane protein was used as a control. All labeled nanorods were transferred to PBS and sonicated for 3 min to suspend the SiNRs into solution. The final working medium concentration of SiNR was about 2.0 × 10^7^ nanorods per milliliter.

### 2.10. Quantitative Analysis on Nanorod Uptake

The cells were seeded in a 24-well plate at a 5 × 10^4^ seeding density. The SiNRs (final working concentration ~2.0 × 10^7^ per milliliter) were added and incubated for 6 h. The live cells were stained with Alexa-594-conjugated WGA and Hoechst for 20 min and then detached by 3 mM EDTA in PBS followed by 4% PFA fixing for 15 min at 37 °C.

Imaging flow analysis was completed using an Amnis ImageStream X Mark II. Channel 0 bright field, SSC, Laser 405, 488, and 561. Cell samples were analyzed at a low speed for best sensitivity. The gating strategy is shown in Figure S4A. The data analysis was performed using Ideas 6.2 and Flowjo VX. To validate the cell-type specific internalization efficiency, we used the statistical module in Ideas software ”spot count” to make an accurate algorithm of internalization. The internalization efficiency is the ratio of the fluorescence intensity inside the cell to that of the entire cell, i.e., internalization% = (cell count with indicated internalization level)/(total cells). This metric was binned to three levels: low (0 < spot count ≤ 3, blue), middle (3 < spot count ≤ 10, yellow), and high (spot count >10, red) (see Figure S4B).

### 2.11. Immunostaining, EGF Binding Assay, Colocalization Assay, and Confocal Microscopy

The cells were seeded on collagen-coated cover slides in a 24-well plate. The internalization protocol was as described above. The 4% PFA fixed cover slides were mounted with antifade reagent (Thermo, Waltham, MA, USA).

For the ATP1A1 immunostaining, the SiNR (non-fluorescence conjugated) or cells were blocked in 1% BSA PBS-Tween20 buffer after 4% PFA fixing. We used 1:200 dilution of ATP1A1 Polyclonal Antibody (bs-9570R, Bioss, Beijing, China), followed by Alexa Fluor 488 goat anti-rabbit antibody staining. After washing with PBS-Tween20 3 times, the coverslips were mounted with antifade reagent for confocal microscopy.

For the EGF binding assay, 10 μg human EGF (Z00333-50, GenScript, Nanjing, China) were labeled with Cy3-SE using a Super-n-Stain™ Antibody Labeling Kit (S601108-s, Us everbright, Suzhou, China). MP coated SiNR or uncoated SiNR were incubated with 5 μg/mL labeled EGF at 37 °C for 2 h. After washing with PBS-Tween20 3 times, the coverslips were mounted with antifade reagent for confocal microscopy.

For the colocalization assay, the cells were seeded on collagen-coated cover slides in a 24-well plate. The internalization was examined as described. The cells were then incubated with Lyso-Orange (1:500 dilution, ab176827, Abcam, Cambridge, England) for 20 min combined staining with Alexa-633-conjugated WGA and Hoechst. The 4% PFA fixed coverslip was mounted onto a slide with antifade reagent (Thermo, U.S.).

Photographs were performed using a Leica SP8 laser confocal microscope at the National Center for Protein Science in Shanghai.

### 2.12. Total RNA Isolation and qRT-PCR Analysis

The total RNA was extracted using a Total RNA Isolater (Vazyme, Nanjing, China) according to the manufacturer’s instructions. The RNA was collected, and 1 μg of RNA was reverse transcribed into cDNA with HiScript II Q RT SuperMix (Vazyme, Nanjing, China) according to the manufacturer’s instructions. The real-time PCR analysis was performed using the LightCycler (Roche, USA) system with ChamQ SYBR qPCR Master Mix (Vazyme, Nanjing, China). The primer sequences for qRT-PCR procedures are listed in the Supporting Information Table S3.

### 2.13. Small Interfere RNA Transfection and Chemical Inhibitors Studies

The *ATXN2*-targeting siRNA and negative control siRNA were purchased from Genepharma (Shanghai, China). The detail sequence is shown in Table S4. The transfection of siRNA was performed using INTERFERin (Polyplus, USA) according to the manufacturer’s protocol. Briefly, 2.5 × 10^4^ Panc-1 cells per well were seeded in 12-well plates and transfected the next day with a 100 nM final concentration of siRNA using 2 μL INTERFERin. The cells were harvested 48 h after transfection for real-time PCR analysis and western blot assay.

For the imaging cytometry and chemical inhibitors assay, Panc-1 cells were seeded (8 × 10^4^ cells) in 6 cm tissue culture plate and then allowed to adhere overnight. The cells were transfected the next day with a 100 nM final concentration of siRNA using 20 μL INTERFERin. The cells were cultured 48 h before the internalization assay. Inhibitors were used and added to the cells at the concentrations described in Figure 8E. The cells were incubated with Panc-PDX@SiNR and indicated inhibitors for 6 hrs. The internalization protocol was as described above.

### 2.14. Statistic Method

The data were expressed as mean ± SD based on at least three independent experiments. Statistical analysis used the Student’s *t*-test. The differences were significant with a *p* value <0.05.

## 3. Results

### 3.1. Fabrication of Size-Controllable SiNR Array

**Figure 1:**
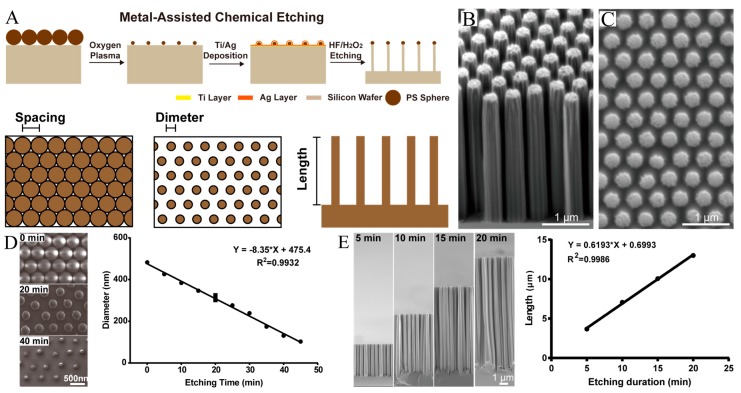
Fabrication and characterization of the SiNR array. (**A**) Scheme of chemical etching process for fabricating SiNRs. The spacing, dimeter, and length of the nanorods are controllable. (**B**) SEM image of SiNR array (45° title view). The length of nanorods are uniform. (**C**) SEM image of SiNR array (top view). The diameters of nanorod are uniform. (**D**) The left panel is selected SEM images of PS spheres after oxygen plasma etching (0 min, 20 min, and 40 min). The right panel shows the correlation between the diameter of PS sphere and the etching time of oxygen plasma. A significant negative correlation was observed between the diameter and the etching time (R^2^ = 0.9932, *p* < 0.0001). (**E**) The left panel presents SEM images the SiNR array (sectional view) after chemical etching (5 min, 10 min, 15 min, and 20 min). The right panel shows the correlation between the length of SiNR array and the etching time of oxygen plasma. A significant positive correlation was observed between the diameter and the etching time (R^2^ = 0.9986, *p* < 0.0001).

The SiNR fabrication techniques can be classified into the following two categories: bottom-up and top-down techniques [40]. The bottom-up method originated from the vapor-liquid-solid mechanism of Si nanorod growth [41], but its scale-up capacity is very limited. Top-down fabrication uses an etching process such as wet chemical etching or reactive ion etching. On the basis of previous methods [13,39], we introduced a facile chemical etching method for SiNR fabrication. Figure 1A shows that a single layer of polystyrene (PS) spheres is formed as a template via self-assembly on the silicon substrate. The following oxygen plasma etching process is used to shape the diameter of the PS spheres: a 5 nm titanium film and 30 nm silver film are sequentially e-beam processed and thermal evaporated onto the silicon substrate as the catalyst. A Ti/Ag film with a hexagonal array of holes is formed due to the PS monolayer mask. Subsequently, an etching step is conducted in a mixture of deionized water, HF, and H_2_O_2_. The Ag film catalyzes the etching of silicon beneath it. During the etching process, the “walls” of the honeycomb are gradually etched away, and the remnant silicon forms a nanorod array. Finally, the silver film and PS spheres are eliminated by HNO_3_ and chloroform, respectively. Figure 1B,C show SEM images of silicon nanorod arrays from a tilted view (ca. 45°) and a plane view, respectively. The length of the fabricated nanorods is 3.495 ± 0.108 μm, and the diameter is 0.390 ± 0.009 μm. The results indicate that the nanorod distributes uniformly with a homogenous length and diameter.

Both the diameter and the length of the SiNR are key parameters that control size. The diameter of the SiNR is determined by the size of the PS sphere mask. The etching time is a precisely-controlled parameter (Figure 1D). Therefore, we studied the change in the diameter of the PS spheres as a function of oxygen plasma etching time. In Figure 1D, the left panel shows selected SEM images of the PS spheres after various etching times. The right panel shows three parallel samples per measurement for a total of 10 time points (0–45 min); linear regression was performed to verify the correlation between the etching time and the PS sphere diameter. The modeling result is significant: The R-squared value reaches 0.9932, and the *p*-value is <0.0001. These metrics demonstrate that the diameter of the PS sphere can be precisely controlled via etching time.

The length of the SiNR arrays is also related to the duration of etching after silver film evaporation. Figure 1E shows cross-sectional SEM images of SiNRs fabricated with etching times of 5, 10, 15, and 20 min yielding nanorods with lengths of ca. 3.67, 7.05, 10.5, and 12.99 µm, respectively. The length of the nanorods varies linearly with the duration of the etching process and provides good control over the length of the nanorod arrays. This method is easy and practical for SiNR size control.

Three sizes of SiNR were fabricated to investigate the SiNR internalization. Detailed morphology of SiNRs was observed by SEM (Figure S1F–H). As shown in Table 1, the large size (L-size) has the longest length, 3.495 ± 0.181 μm, and the middle size (M-size) is 2.229 ± 0.049 μm long. The L-size and M-size have the same diameter (0.409 ± 0.010 μm). The small size (S-size) is 0.105 ± 0.011 μm wide and 0.656 ± 0.126 μm long and its volume is about 1/54 of the M-size SiNR.

### 3.2. Nanofabrication of SiNR Coating with Membrane Protein

**Figure 2:**
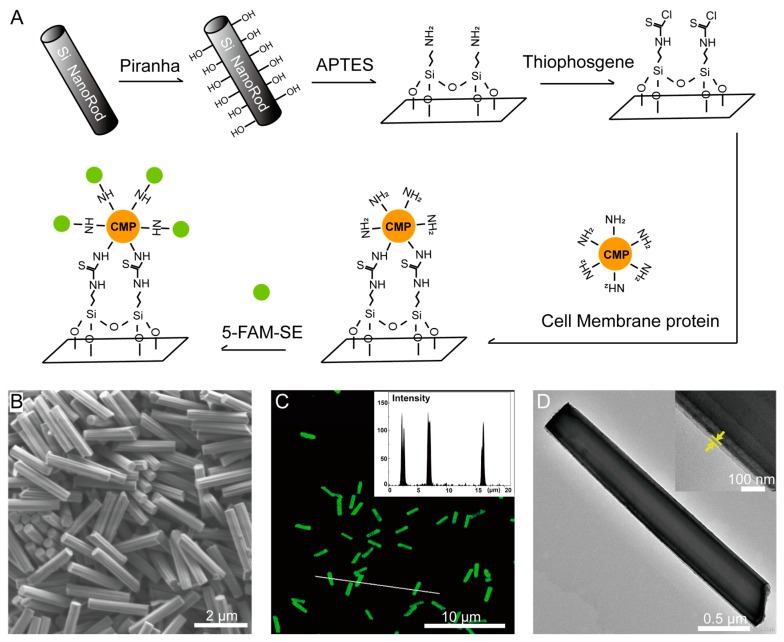
Coating and characterization of silicon nanorods: (**A**) Scheme of SiNR fabrication, (**B**) SEM images of the SiNR, (**C**) Silicon nanorods labeled with fluorescence (SiNR-CMP-FAM), and (**D**) transmission electron microscope image of a single SiNR with CMP (from Hela cell). Scale bar indicates 500 nm. Detailed TEM image of CMP (inner picture), yellow arrow indicates CMP layer. Scale bar stands for 100 nm.

Different physiochemical factors influence the nanoparticle internalization such as surface charge, shape, size, and bioconjugate moiety [4,5,14,42,43,44,45,46,47,48,49,50,51]. After preparing SiNR arrays of three different sizes, the SiNR were obtained by carefully scraping them off the array. We subsequently used surface modification via a cell membrane protein. Figure 2A shows the scheme to fabricate SiNR coatings with the same amount of cell membrane protein (see Materials and Methods Section 2.9). Five different cell membranes were used to coat the SiNRs using the same total protein starting concentration.

After SiNR nanofabrication and protein coating, the 5-FAMSE fluorescence was linked to a protein coating layer. We have already described the controlled fabrication and here focus on a unified size for testing internalization efficiency as a function of coating. Images of the SiNR, as shown in Figure 2B, had a uniform size (408 ± 10 nm in diameter and 2229 ± 49 nm in length). The confocal images indicate homogeneous fluorescence intensity for each SiNR in Figure 2C. The coating membrane proteins (CMP) on the surface of SiNR were investigated by TEM (Figure 2D and Figure S1A–E), and the average thickness of the CMP layer was 19.09 ± 0.47 nm. Dynamic light scattering (DLS) was performed to analyze the particle size distribution of SiNR. Figure S1F shows SiNR has good homogeneity after CMP coating.

### 3.3. Proteomics Analysis of Coating Membrane Proteins Derived from Five Cancer Cell Lines

**Figure 3:**
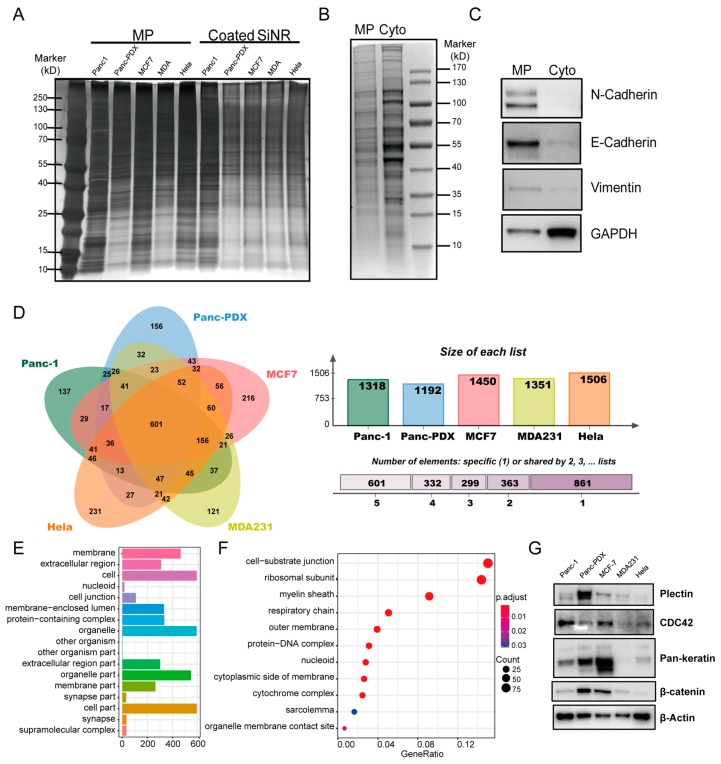
Proteomics analysis of five cell membrane extracts. (**A**) Silver staining of membrane protein lysate and CMP-coated SiNR PAGE with 20 μL protein loading. Panc-1 cell membrane extraction and cytoplasmic extraction were confirmed by silver staining (**B**) and western blot (**C**). N-cadherin and E-cadherin are cell junction markers, vimentin is a cytoskeleton protein, GAPDH is a metabolic enzyme located in the cytoplasm. (**D**) Venn plot of the proteins identified from the five types of cell membrane extracts. The center part of the plot stands for the 601 common proteins in all five cell types. The outer single-color parts are unique proteins which belong to each single type cell. The top right panel shows the total protein number of each cell type. The buttons in the right panel are different shared proteins among the five types of cell membrane extracts. (**E**) The cellular component group has 601 common proteins; 50% of the proteins are associated with the extracellular region and 16.7% proteins belong to the cell junction gene set. (**F**) GO enrichment result of the 601 common proteins. The focal adhesion term is enriched with a significant *p*-value <$1\times 10^{-9}$. (**G**) Selected protein targets from the common 601 proteins in the five cell types: Plectin, CDC42, β-catenin, and β-actin.

Using cell membrane proteins as a surface grafting moiety can be a practical internalization strategy, however, the exact composition and variation of the proteins in the membrane are often unknown [7]. To verify the membrane protein coating on SiNR, all five SiNR with different coating were boiled in loading buffer for PAGE followed by silver staining. The protein coating on the SiNR surface had the same molecular weight pattern as that in the membrane protein lysate (Figure 3A and Figure S2). We also employed an antibody of the cell membrane marker ATP1A1 for immunostaining [52]. The confocal images (Figure S3A–C) showing ATP1A1 staining on MP-coated SiNR indicate that the MP is coated on the SiNR surface. To confirm the biological function of MP after the coating process, we used the Cy3-labeled human EGF to incubate with MP-coated SiNR. The confocal pictures (Figure S3D) show the fluorescence signal of human EGF was merged with the MP-coated SiNR but not the uncoated SiNR. This colocalization demonstrated that the EGFR proteins on SiNR remain functional in binding to its specific ligand.

We analyzed Panc-1 to validate that the protein extraction was indeed from the cell membrane. N-cadherin and E-cadherin are proteins that anchor the plasma membrane and act as cell junction markers [53] and they were highly expressed in the Panc-1 membrane extract (Figure 3B,C). Vimentin is a cytoskeleton protein that acts as an intermediate filament. It was present on both the membrane extract and the cytoplasmic lysate. Protein gel sets derived from the above setting were used for high-resolution LC-MS/MS analysis for protein identification and characterization. On the basis of this unique peptide match, the final proteomic data (Supporting Information Section 2) were used for matching in the uniprot (*Homo sapiens*) database.

We identified 1318 proteins from Panc-1, 1192 from Panc-PDX, 1450 from MCF-7, 1351 from MDA231, and 1506 from Hela cells. A Venn diagram was employed to compare the variation among the five cell membrane proteins, and a full list of uniprot proteins identified from each cell membrane was uploaded to Jvenn (http://jvenn.toulouse.inra.fr). The results indicate that 601 proteins were present in all five cell lines, and 861 proteins were present in only one of the five lines (Figure 3D). Of the 601 shared proteins, two dominant clusters are proteins associated with the extracellular region (50%) and proteins belonging to cell junction function (16.7%). In Figure 3F, GO enrichment shows that the cell substrate junction term is the most enriched segment with a significant *p*-value (<$1\times 10^{-9}$). This implies that the cell–cell contact and cell-substrate adhesion proteins are key proteins in nanorod coating for internalization.

To further investigate the protein expression diversity among the five cell membranes, a few selected proteins were Western blotted for validation (Figure 3G). Plectin is a biomarker for invasive pancreatic ductal adenocarcinoma [54,55]. It was highly expressed in the Panc-PDX cells which was the primary tumor sample. Pan-keratin is an epithelial marker of epithelial malignancies [56]. It was highly expressed in Panc-PDX and MCF-7 cells. The expression pattern of β-catenin was similar to pan-keratin, i.e., it was enriched in epithelial-like Panc-PDX and MCF-7 cells. Meanwhile, MCF-7 and Panc-1 had higher levels of Cdc42. These are a Rho family member of GTPases [57]. All of these results suggest that cell membrane proteomics are cell-type dependent.

### 3.4. SiNR Internalization Efficiency Is also Determined by Target Cells

**Figure 4:**
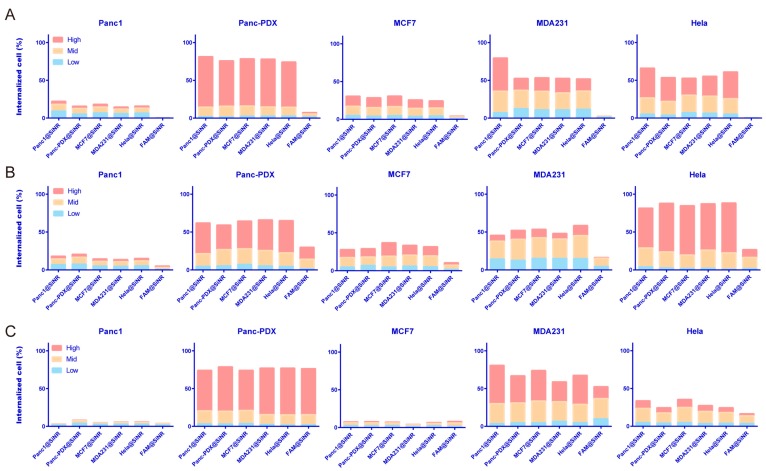
Cell distribution and internalization of membrane-coated SiNRs. The amount of SiNR internalized per cell was measured via an index of spot count: low internalization 0–3, middle internalization 3–10, high internalization >10. The cell distribution of different SiNR internalizations are shown in the bar plot. The FAM-linked SiNR served as a control. (**A**) L-size SiNRs were incubated with the indicated cell line. (**B**) M-size SiNRs were incubated with indicated cell line. (**C**) S-size SiNRs (100 nm diameter) were incubated with the indicated cell line. The FAM-linked SiNR served as a control.

The SiNR uptake is likely determined by coating and the intrinsic properties of target cells. To further characterize the interactions between coated SiNR and the target cells, we conducted quantitative and consecutive analysis of SiNR internalization efficiency in each of the five cancer cell lines using the AMNIS ImageStream system.(Figure S4A,B). Statistics on the internalization efficiency are shown in Figure 4, Figure S4H–I, and Table S1 for the SiNR coatings with different membrane proteins. We used the following index of the spot count to represent the SiNR amount internalized in a particular cell (Figure S4B): low internalization 0–3, middle internalization 3–10, and high internalization >10. The L-size and M-size SiNR coatings with different cell membranes show enhanced internalization efficiency as compared with non-MP coated SiNR (FAM@SiNR) in all cell types (Figure 4A,B). However, the MP coating does not improve the S-size SiNR internalization efficiency.

**Figure 5:**
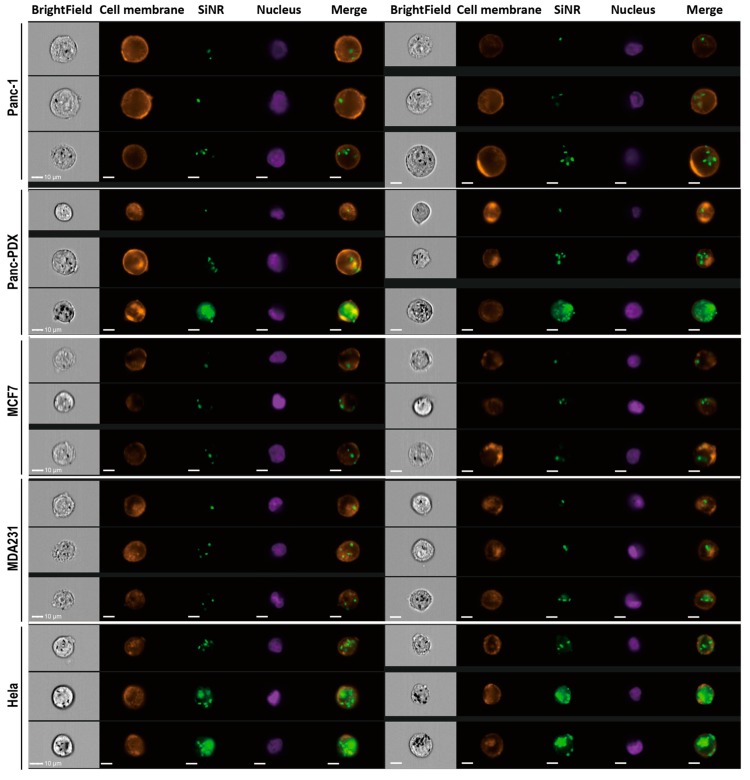
Imaging cytometry analysis of the five types of cells coated on M-size SiNRs, including the Panc-1 membrane protein. Six cytometry images from the indicated cell lines represent the morphological and fluorescence features of SiNR uptake. The SiNR can be observed in both bright field and FAM (green) channels. Hoechst (purple) indicates the cell nucleus for the cytometer. Alexa-594 (orange) conjected WGA staining shows the cell membrane. Cell membrane/SiNR merged channel indicates the location of the SiNR inside the cell. The images are as follows: bright field, cell membrane, SiNR, nucleus, and merge of cell membrane and SiNR. Scale bar represents 10 µm.

The internalization efficiency is different across cell types. Thus, we performed a quantitative analysis by ImageStream. The M-size SiNR average internalization of the five investigated cells are Hela (78.72%) > Panc-PDX (58.83%) > MDA231 (45.74%) > MCF7 (28.18%) > Panc-1 (14.44%). This ranking order corresponds well with ImageStream on internalization in Figure 5 and Figure S4D–H, which detail the internalization efficiency of all five cell types. The histogram plot of the M-size SiNR fluorescence intensity (MFI) in Figure S4C shows the cell-type specificity on internalization. The rank order was Hela>Panc-PDX > MCF7 > MDA231 > Panc-1. The MFI in Hela ($9.44\times 10^{4}$) is the highest and is twice as high as Panc-PDX ($4.722\times 10^{4}$).

**Figure 6:**
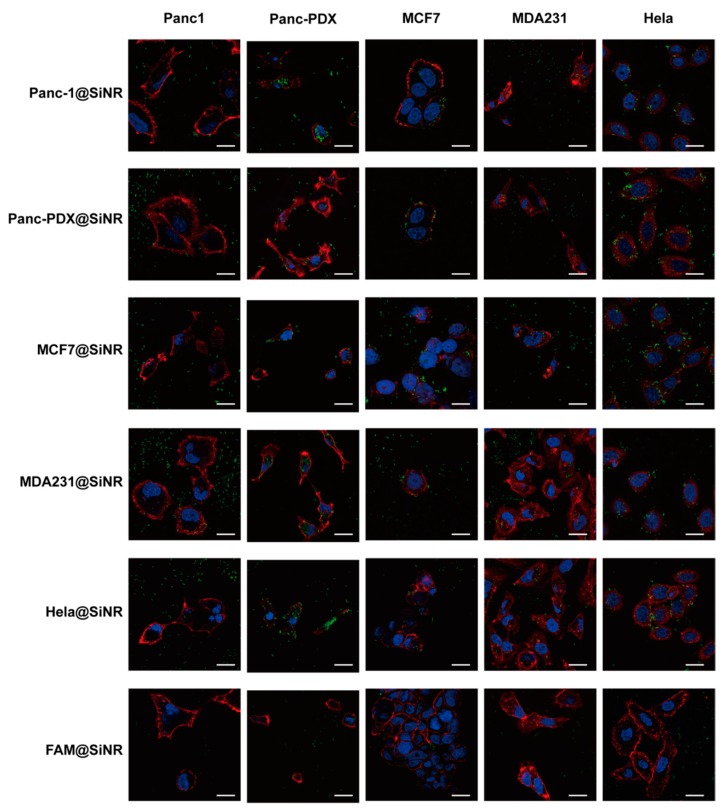
Cell morphology and SiNR location by confocal laser microscopy. The SiNR with FAM (green), cell nucleus stained with Hoechst (blue), Alexa-594 conjected WGA staining cell membrane (red) were observed. The scale bar in white stands for 20 μm. The FAM only linked SiNR was used as a control.

This improved internalization efficiency expands from the homolytic coating proteins to the heterolytic proteins (Figure 4, Figure S4I,J, and Table S1). This result is confirmed by cytometry as well as confocal images. Figure 6 and Figure S5 show a detailed SiNR internalization process with different coatings across the five type cells. We used z-stack scanning of each type of single cell to identify the internalized SiNR. In Figure S5A, each picture crosshair points the SiNR presented near nucleus inside the cell body (cell membrane). Meanwhile, the lysosome fusion with MP-coated SiNR was observed in MCF7 cell (Figure S5B). In Figure 6, the middle sections with observed nucleus were chosen to capture internalized SiNR near nucleus and inside the membrane staining. The SiNRs coating with membrane proteins are significantly aggregated inside the Hela and Panc-PDX cells and less aggregated inside MCF7 and MDA231 cells. For Panc-1, most SiNR spread outside the cells, and a very small number of SiNRs were internalized. This is because Panc-1 cells have the lowest ranking and have an intrinsic mechanism at the molecular level. The internalization efficiency is cell-type dependent.

### 3.5. Gene Ontology on Cancer Cell Internalization

**Figure 7:**
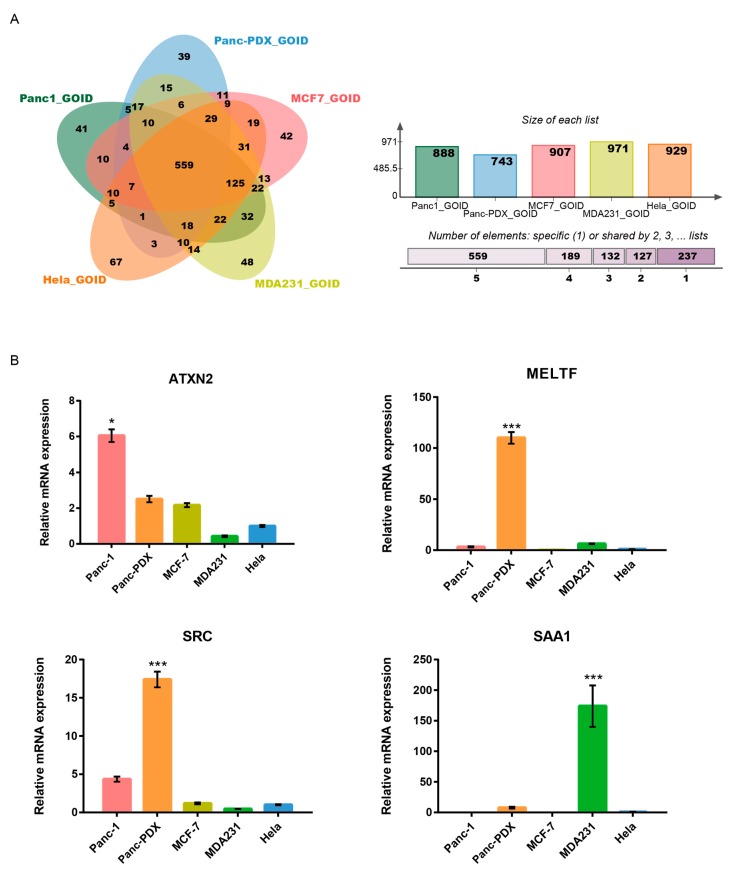
Screening for potential SiNR endocytosis-related genes. (**A**) Venn plot of GO terms enriched from five types of cell membrane proteins. The center part of the plot stands for the 559 common gene sets that are contained in all five types of cells. The overlying parts surrounding the center cover the partial shared gene sets with specific types of cells. The outer single-color parts are unique functional gene sets belonging to each single type cell. The top-right panel shows the total GO terms enriched from every type of cell membrane protein pool. The right panel represents different shared levels of GO terms among the five types. (**B**) Real-time PCR results show *ATXN2*, *MELTF*, *SRC*, and *SAA1* genes. A multiple *t*-test was performed with three biological replicates per group in GraphPad 6 (* *p* < 0.05, *** *p* < 0.001).

To understand the molecular mechanism underlying SiNR internalization in the system, we performed a bioinformatic analysis on the proteomic data of membrane proteins from five cell lines (Supplemental File 2). The GO (Supporting Information Section 2) mapping shows that all identified cell membrane proteins from five specific type can be divided into the following three categories: cellular component (CC), biological process (BP), and molecular function (MF). Figure 7A shows that these five cell types share 559 common GO terms. However, each type takes a different number of unique genes: 41 (Panc-1), 39 (Panc-PDX), 42 (MCF-7), 48 (MDA231), and 67 (Hela). In this research, Panc-1 shows much lower internalization efficiency than the others. The intrinsic cell-type specificity is attributed to this internalization variation. A GO term named “negative regulation of endocytosis” is found only in the gene enrichment result of Panc-1 membrane extracts.

By searching for the potential internalization driver gene (Table 2 and Table 3, and Table S2) with alignment on the list of unique proteins from the cell membrane extraction (Figure 3D), we identified *ATXN2* as a unique gene annotated via “negative regulation of endocytosis” in Panc-1 cells. *ATXN2* is involved in endocytosis [58,59] and it functions as a repressor of the mTOR signal pathway [60]. One interesting question is whether its upregulation is associated with the lowest internalization efficiency of Panc-1. To validate *ATXN2* expression in different cell lines, we analyzed *ATXN2* mRNA levels using RT-RCR. The mRNA level of *ATXN2* in Panc-1 cell is highest among the five cell lines (Figure 7B). This trend is inversely correlated with the nanorod internalization efficiency, suggesting that *ATXN2*-associated down-regulation of endocytosis might be involved in the process. This finding supports the low internalization efficiency in Panc-1. At the same time, two other genes were upregulated in Panc-PDX which is the only primary cell of the five types. One was *MELTF*, a gene that encodes melanotransferrin [61,62] and plays a vital role in cell migration [63]. The other was C-Src, which is anchored at the inner face of the plasma membrane and regulates cell motility and cell-cell adhesion [64,65].

### 3.6. ATXN2 Is a Negative Regulator of Nanorod Internalization

**Figure 8:**
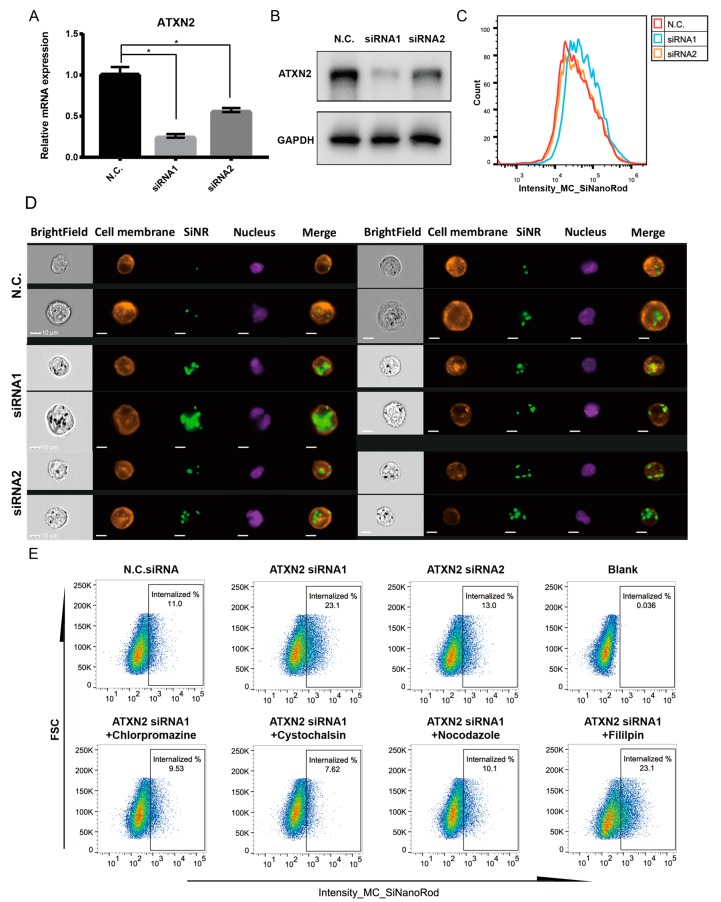
Verification of the role of *ATXN2* in SiNR internalization. (**A**) Real-time PCR for *ATXN2* knockdown validation in Panc-1 cell lines. A multiple *t*-test was performed with three biological replicates per group, * *p* < 0.05. (**B**) Western blot for *ATXN2* knockdown validation in Panc-1 cells. GAPDH was used as loading control. (**C**) Intensity histogram of SiNR in Panc-1 cell. Red line is the negative control; orange line is the siRNA1-transfected cells; blue line is the siRNA transfected cells. (**D**) Four selected imaging cytometry images per population. The panels of one group of images are as follows: bright field, cell membrane, SiNR, nucleus, and merge of cell membrane and SiNR. Scale bar represents 10 µm. (**E**) Spot plot of FSC (forward light scatter) vs. SiNR FAM fluorescence. Panc-1 cells with indicated treatment were incubated with Panc-PDX@SiNR. All cells subjected to analysis were first plotted by FSC vs. SSC (side light scatter), followed by gating for single cell population and then nucleus-stained cells (Hoechst). ”Internalized” gate was set for gating SiNR positive cells based on a blank control (no SiNR). The working concentrations of the chemical inhibitors were chlorpomazine 30 μM, cystochalsin D 500 nM, nocodazole 20 μM, and filipin 40 μg/mL.

To further investigate the role of *ATXN2* in nanorod internalization, we first knocked down *ATXN2* expression in Panc-1 cell with specific interfering RNA (siRNA). As validated with RT- PCR (Figure 8A), siRNA1 and siRNA2 of *ATXN2* inhibited *ATXN2* expression by 77% and 45%, respectively. Meanwhile the Western blot (Figure 8B) also showed the consistent result that the protein levels of *ATXN2* were knocked down by the two siRNAs, respectively. Imaging flow cytometry was then performed to quantify the SiNR cellular uptake in control and *ATXN2* knockdown Panc-1 cells (Figure 8C). The *ATXN2* knockdown from siRNA1 induced more efficient inhibition of *ATXN2* expression and resulted in a greater improvement in internalization efficiency. The expression of siRNA2 was consistently less efficient in *ATXN2* knockdown and led to modest or no improvement of internalization. The single cell images from imaging cytometer (Figure 8D) also confirm the SiNR internalization pattern after the *ATXN2* siRNA treatment. These results support a correlation between *ATXN2* expression and SiNR internalization efficiency.

To understand the mechanisms that *ATXN2* involved in cellular internalization of SiNR, we used chemical inhibitors and low temperature treatment to block the *ATXN2* siRNA-induced SiNR internalization in Panc-1 cell. As Figure S6 shows, the *ATXN2* siRNA1 induced SiNR uptaken was block at 4 °C. The results from low temperature treatments suggest that SiNR internalization affected by *ATXN2* is energy dependent.

The chemical inhibitors are chlorampramazine, a specifically blocker of clathrin-mediated pathway [66]; filipin, a caveolae pathway inhibitor [66]; cytochalasin D which inhibits actin polymerization that is involved in various other endocytic pathways [66]; and nocodazole blocks microtubule polymerization and vesicular transport [45].

The chemical inhibitors were used to test their effects on SiNR internalization. As shown in Figure 8E, *ATXN2* siRNA1 induced a 2.1-fold increase in cells with internalized SiNR, as compare with cells treated with NC scrambled siRNA. In contrast, chlorampramazine, cytochalasin D, and nocodazole, but not filipin, reversed the uptake improvement induced by *ATXN2* siRNA. The results from the inhibitor assays suggest that *ATXN2*-modulated SiNR internalization may involve clathrin, micropinocytosis, and microtubules systems, but not the cavelae pathway in Panc-1 cells.

## 4. Discussion

Silica nanomaterials are safe and biocompatible in vitro and in vivo [12,14,67]. The cell membrane protein coating method is a powerful method to enhance internalization efficiency [6]. Here, we developed a facile method to fabricate silica nanorods with a controllable size, shape, and amount of coating protein from cell membranes for enhanced efficiency uptake. We then determined potential factors that may affect the internalization efficiency.

The diameter and length of SiNR can be precisely controlled by etch time, and three types of SiNRs were fabricated. Our method improved the internalization efficiency via SiNR size optimization. Our fabrication method employed the free -NH_2_ of protein to form covalent bonds with the SiNR surface -OH via APTES and thiophosgene. We believe this is an unbiased method to link all types of cell membrane proteins to a SiNR surface.

We used silver staining, immunostaining, as well as TEM and EGF binding assays (Figure 3A, Figures S1 and S3) to demonstrate that the cell membrane proteins were indeed linked to the SiNR surface. The free amine-mediated coating method used a proportional number of proteins coated to the nanorods. Due to the limited protein extracts available for coating the SiNR, we used direct cell membrane protein extraction to investigate the cell membrane proteomic fingerprints of the five cell lines.

The Amnis system is an establish method for internalization detection [68,69,70]. A practical protocol for eliminating the cells with background labeling and identifying specific internalization was published in 2012 [70]. We followed the protocol to identify the SiNR internalization, and the details are shown in Figure S4A. As confocal microscopy can provide more details on the SiNR uptake, the pictures from Z-stack scanning proved MP-coated SiNR was present inside the cell body in a three-dimensional view. Moreover, our lysosome colocalization assays indicate that the MP-coated SiNR internalization may be relative to the canonical endocytosis pathway [71].

Hela, Panc-1, Panc-PDX, MDA321, and MCF7 cells were used in this study to investigate the internalization efficiency. The FACS data showed that the high SiNR uptake in target cells led to a higher total internalized cell rate and a greater percent cell distribution (Figure 4). We found that the membrane coating significantly improved the L-size and M-Size SiNR internalization efficiency in all tested cells. This improved internalization efficiency holds for both homolytic and heterolytic proteins. However, this coating did not enhance the S-Size SiNR internalization which may be due to size-related different endocytosis mechanisms. For example, macropinocytosis can take about 2 μm particles but clathrin-mediated endocytosis prefers particles <300 nm [72]. This issue will be further investigated in future work.

To understand the mechanism underlying the altered internalization, we compared the proteomics data of the five cell membrane proteins and identified 601 proteins that are known as having cell substrate adhesion properties. These proteins include N-cadherin, E-cadherin, and integrin. These might contribute to the surface recognition and internalization. The homolytic or heterolytic SiNR coatings have the same improvement which may be due to these surface proteins.

In contrast to coating sources, the internalization efficiency of the target cells varied among the tested cell lines across all three sizes of SiNR. This motivated us to identify potential cellular factors that may affect nanorod internalization. Integration of the proteomics data and the internalization efficiency data suggested an association between the lowest internalization efficiency in Panc-1 cells and *ATXN2* upregulation in these cells. The improved internalization efficiency in Panc-1 cells with *ATXN2* knockdown provided specific evidence supporting *ATXN2* as a negative regulator of nanorod internalization.

Previous studies of nanoparticle internalization have provided specific guidance on the mechanisms of endocytosis with inhibitors and temperature control [45,73,74]. To further understand the mechanisms of SiNR uptake in context with *ATXN2* function, we used chemical inhibitors to explore the pathways the *ATXN2* may participate in. The working concentrations of inhibitors were chosen based on a previous study [45]. As shown in Figure 8E, we showed that chlorampramazine, cytochalasin D, and nocodazole, but not flilipin, partially revered *ATXN2* siRNA1 knockdown-induced MP uptake. The results from the inhibitor assays indicate that the *ATXN2* may participate in Panc-1 cell uptake the MP-coated SiNR, including clathrin, micropinocytosis, and microtubules, however, not the cavelae pathway. Our results highlight the intrinsic properties of the target cells that modulate nanocarrier uptake. Our data provide clues to further identification of novel factors that may affect nanoparticle internalization to cancer cells.

In summary, we developed a facile method of silicon nanorod fabrication with controllable sizes. We found that coating the nanorods with cancer cell membrane proteins significantly enhanced the internalization of the nanocarriers. Using proteomics and bioinformatics approaches, we identified proteins that might facilitate the internalization. More importantly, our results highlighted the differences among different target cells in nanorod uptake and identified *ATXN2* as a negative regulator of nanorod internalization. This was further supported by bioinformatic analysis and specific functional validation with siRNA experiments. These data collectively advance our understanding of factors that affect nanocarrier internalization and provide clues to further improve internalization efficiency.

## Figures and Tables

**Table 1:** Size parameters of the three types of SiNRs.

SiNR	Diameter (μm)	Length (μm)
L-size	0.412 ± 0.027	3.495 ± 0.181
M-size	0.409 ± 0.010	2.229 ± 0.049
S-size	0.105 ± 0.011	0.656 ± 0.126

**Table 2:** Potential genes that may affect the SiNR uptake.

Cell line	Genes
**Hela**	***GNAI1 CAV2 SYAP1 LYN JAK1***
MDA231	***SAA1 LOXL2 VTN AHI1 HIP1 THBS1 TGFB2 ITGA5 FAM129B EIF3E***
PDX	***MELTF SRC***
MCF7	***BCL2 GSK3B BAX RHOT2***
Panc-1	***ATXN2 VASP ARPC5L ARPC5 DCTN1 PXN LIMA1 MYH10***

**Table 3:** Cell membrane associated GO terms unique to each cell type.

Cell Type	GO ID	GO Term	Adjust *p*-Value
Panc-1	0001725	stress fiber	6.87 × 10^−4^
	0003779	actin binding	1.06 × 10^−4^
	0005916	fascia adherens	7.08 × 10^−4^
	0007015	actin filament organization	7.46 × 10^−4^
	0008064	regulation of actin polymerization or depolymerization	1.36 × 10^−3^
	0008154	actin polymerization or depolymerization	1.78 × 10^−3^
	0030041	actin filament polymerization	4.13 × 10^−3^
	0030833	regulation of actin filament polymerization	2.83 × 10^−3^
	0030838	positive regulation of actin filament polymerization	2.86 × 10^−3^
	0045806	negative regulation of endocytosis	5.65 × 10^−3^
	0051495	positive regulation of cytoskeleton organization	2.49 × 10^−3^
	0097517	contractile actin filament bundle	6.87 × 10^−4^
	1902903	regulation of supramolecular fiber organization	4.18 × 10^−3^
	1902905	positive regulation of supramolecular fiber organization	4.21 × 10^−4^
Panc-PDX	0031579	membrane raft organization	5.72 × 10^−3^
	0034446	substrate adhesion-dependent cell spreading	1.90 × 10^−3^
	0035635	entry of bacterium into host cell	7.88 × 10^−3^
	1900024	regulation of substrate adhesion-dependent cell spreading	8.61 × 10^−4^
MCF-7	1905710	positive regulation of membrane permeability	5.32 × 10^−5^
MDA231	0019898	extrinsic component of membrane	6.14 × 10^−3^
	0051015	actin filament binding	4.10 × 10^−3^
	0005178	integrin binding	3.51 × 10^−3^
	0005884	actin filament	1.13 × 10^−3^
	0006898	receptor-mediated endocytosis	1.93 × 10^−3^
	0050840	extracellular matrix binding	8.92 × 10^−3^
	0033627	cell adhesion mediated by integrin	7.46 × 10^−3^
	0048259	regulation of receptor-mediated endocytosis	2.20 × 10^−3^
	0048260	positive regulation of receptor-mediated endocytosis	1.96 × 10^−3^
Hela	0031234	extrinsic component of cytoplasmic side of plasma membrane	4.50 × 10^−3^
	0044406	adhesion of symbiont to host	5.66 × 10^−3^

## Supplementary Materials

Supplementary materials can be found at https://www.mdpi.com/2073-4409/8/8/931/s1. Supplemental File 1: Supplementary figures and tables. Supplemental File 2: Identified proteins list from LC/MS. Supplemental File 3: EnrichGO result list.
