# Supplementary material for: Internalization Characterization of Si Nanorod with Camouflaged Cell Membrane Proteins Reveals ATXN2 as a Negative Regulator
Source: Cells. 2019 Aug 19;8(8):931. doi: 10.3390/cells8080931 (PMC6721741; doi:10.3390/cells8080931)
Supplement: Supplementary file 1 [file cells-08-00931-s001.zip › cells-552113-supplementary Proofreading.pdf]

Supplementary Material

Internalization Characterization of Si Nanorod with Camouflaged Cell Membrane Proteins Reveals *ATXN2* as a Negative Regulator

Yi Lu [1,2,†], Jing Dai [2,3,4,†], Na Kong [2], Jianghuai Liu [1], Jinkang Gong [2] and Yuan Yao [2,*]

[1] State Key Laboratory of Pharmaceutical Biotechnology and Ministry of Education Key Laboratory of Model Animals for Disease Study, Model Animal Research Center of Nanjing University, Nanjing 210061, China
[2] School of Physical Science and Technology, ShanghaiTech University, 393 Middle Huaxia Road,, Shanghai 201210, China
[3] Shanghai Institute of Ceramics, Chinese Academy of Sciences, Shanghai 200050, China
[4] University of Chinese Academy of Sciences, Beijing 100049, China
* Correspondence: yaoyuan@shanghaitech.edu.cn
† These authors contributed equally to this work.

Keywords: silicon nanorod; cell membrane proteins; cancer; internalization efficiency; ATXN2

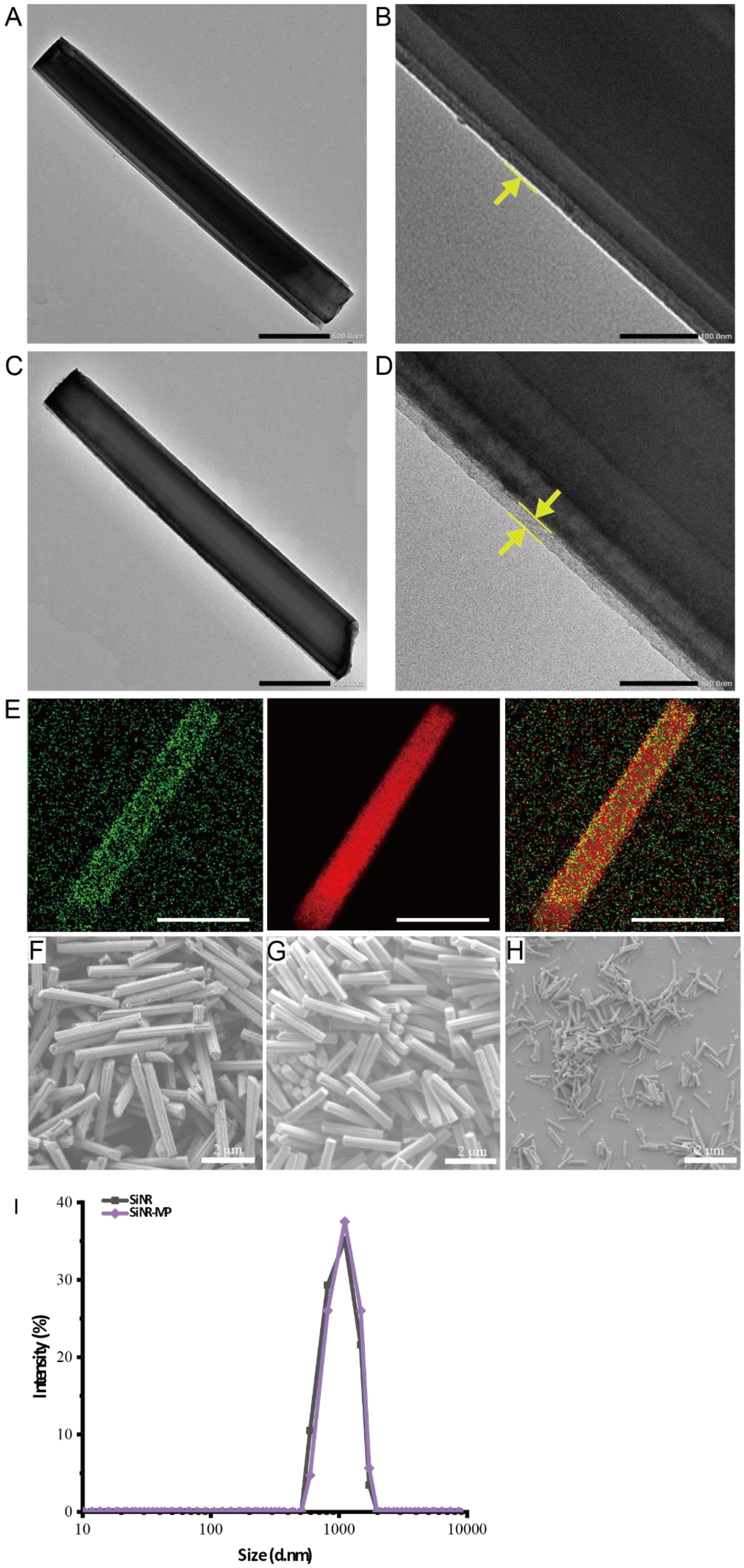
A
B
500.0nm
100.0nm
C
D
E
F
G
H
2 μm
2 μm
2 μm
I
SiNR
SiNR-MP
Intensity (%)
Size (d.nm)
40
30
20
10
0
10
100
1000
10000

Figure S1. (**A–D**) Transmission electron microscope image of the SiNR without MP coating (**A, B**) and SiNR with Hela MP coating (**C, D**). Detailed image of SiNR edge without (**B**) and with Hela MP coating (**D**). (**E**) EDS elemental mapping: Left panel nitrogen mapping, middle panel silicon mapping, right panel merge of nitrogen and silicon mapping. (**F–H**) SEM pictures of three size SiNR, L-size (**F**), M-size (**G**), S-size (**H**). (**I**) Dynamic light scatter analysis of the SiNR with (purple) and without (black) MP coating.

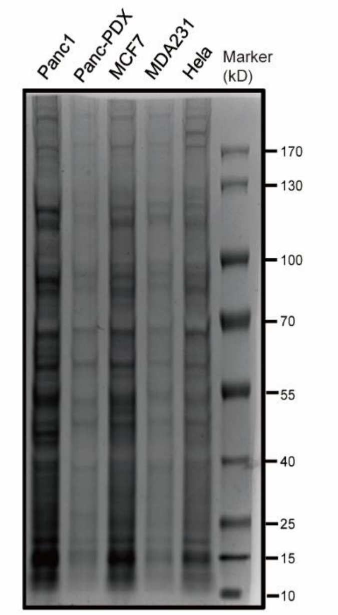

Figure S2. Coomassie blue staining of five cell membrane extracts after SDS-PAGE, 20 μL protein sample loading.

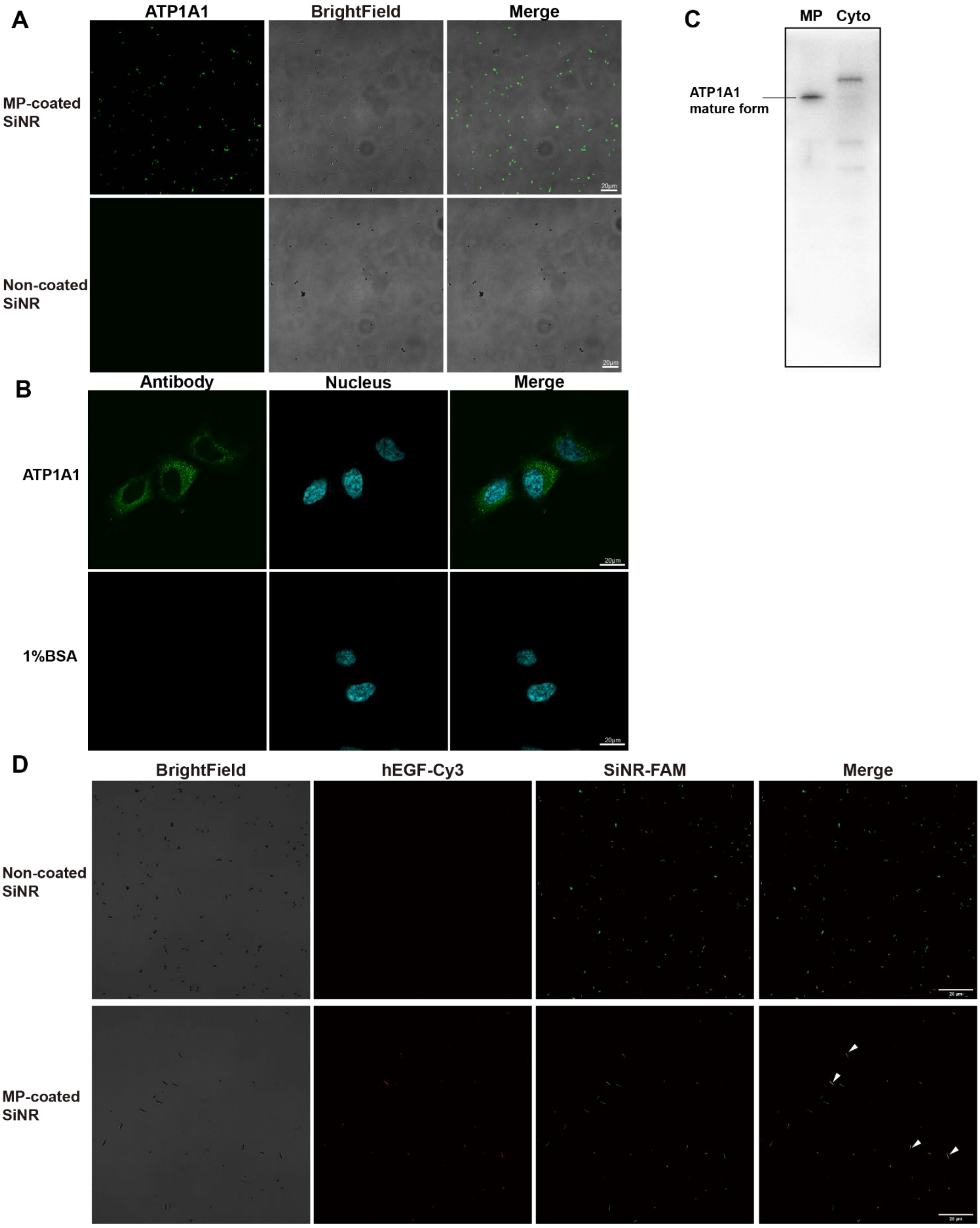

Figure S3. Validation of cell membrane proteins on the coated SiNR and biological function. (A) Confocal microscopy images of SiNR ATPA1 immunostaining. Green fluorescence signal indicates ATPA1 antibody staining, and BF channel shows SiNR. Upper panel: SiNR-coated MP (Panc-1 cell extracted). Lower panel: SiNR without MP coating as a control group shows no ATPA1 signal. (**B**) 293T cell immunostaining; ATPA1 staining (green); Hoechst shows the nucleus (cyan). Scale bars represent 20 μm. (**C**) Western blot shows ATPA1 in a mature form and is mainly in the cell membrane (MP, extracted from Panc-1 cells). (**D**) Cy3 labeled human EGF (red) was incubated with Panc1@SiNR or non-coated SiNR (green) at 37 ^{0}C for 2 h, the white arrows point colocalization (orange) of the EGF signal and Panc1@SiNR in the merge panel. Scale bars represent 20 μm.

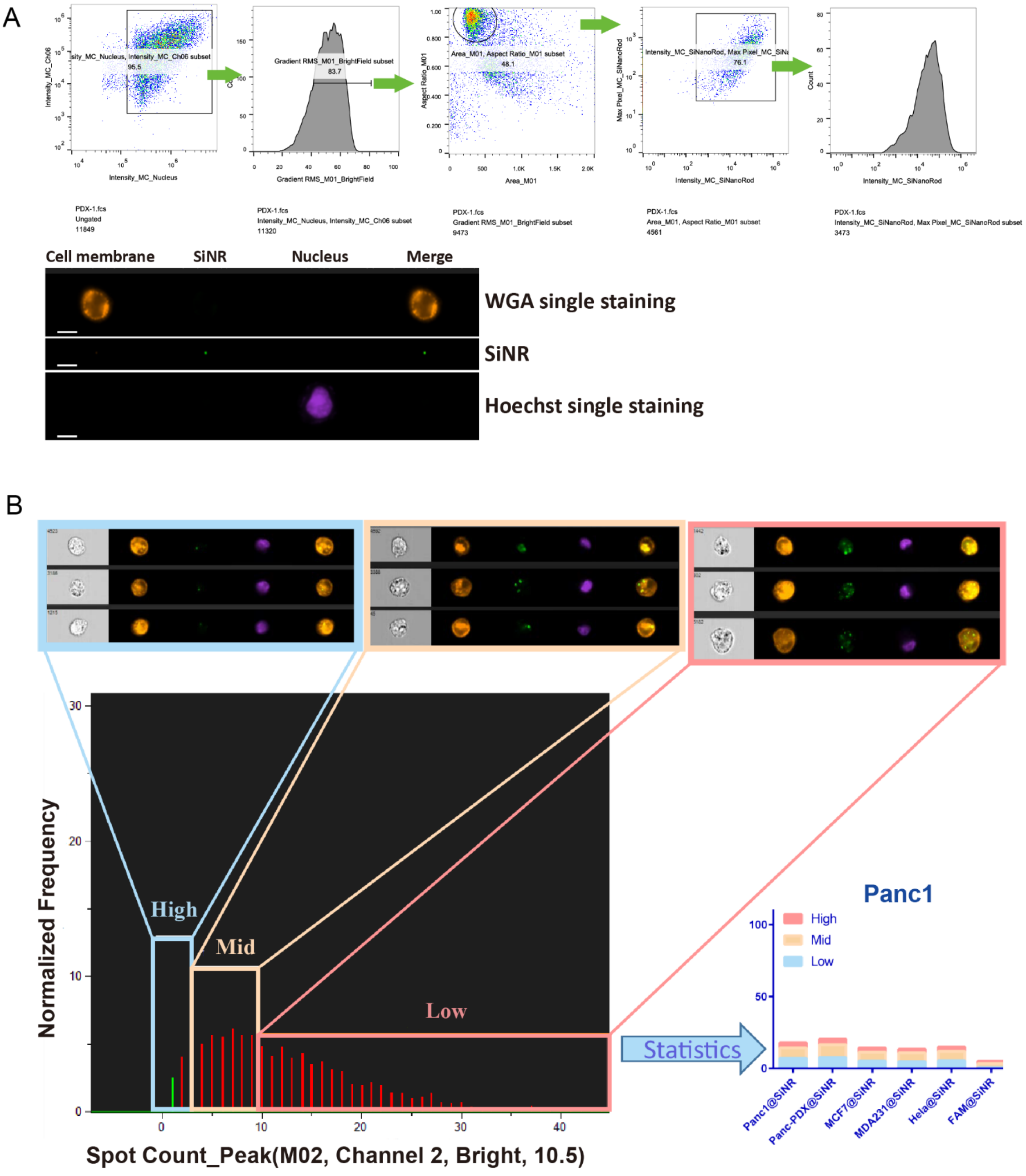

Figure S4AB. Imaging stream cytometry analysis. (**A**) Upper panel shows the gating strategy. First gating is the nuclear staining. Second gating is brightfield focused cells. The third gating is a single cell define via aspect ratio (1 = Round). The fourth gating is a positive signal from SiNanoRods (FAM channel). Lower panel shows selected images from single color samples for compensation. All fluorescence signals are specific to indicated objects: WGA-membrane, FAM-SiNR, and Hoechst-nucleus. The scale bar is 10 μm. (**B**) Diagram for the cell distribution of SiNR internalization according to the spot count index: low internalization 0–3, middle internalization 3–10, and high internalization >10.

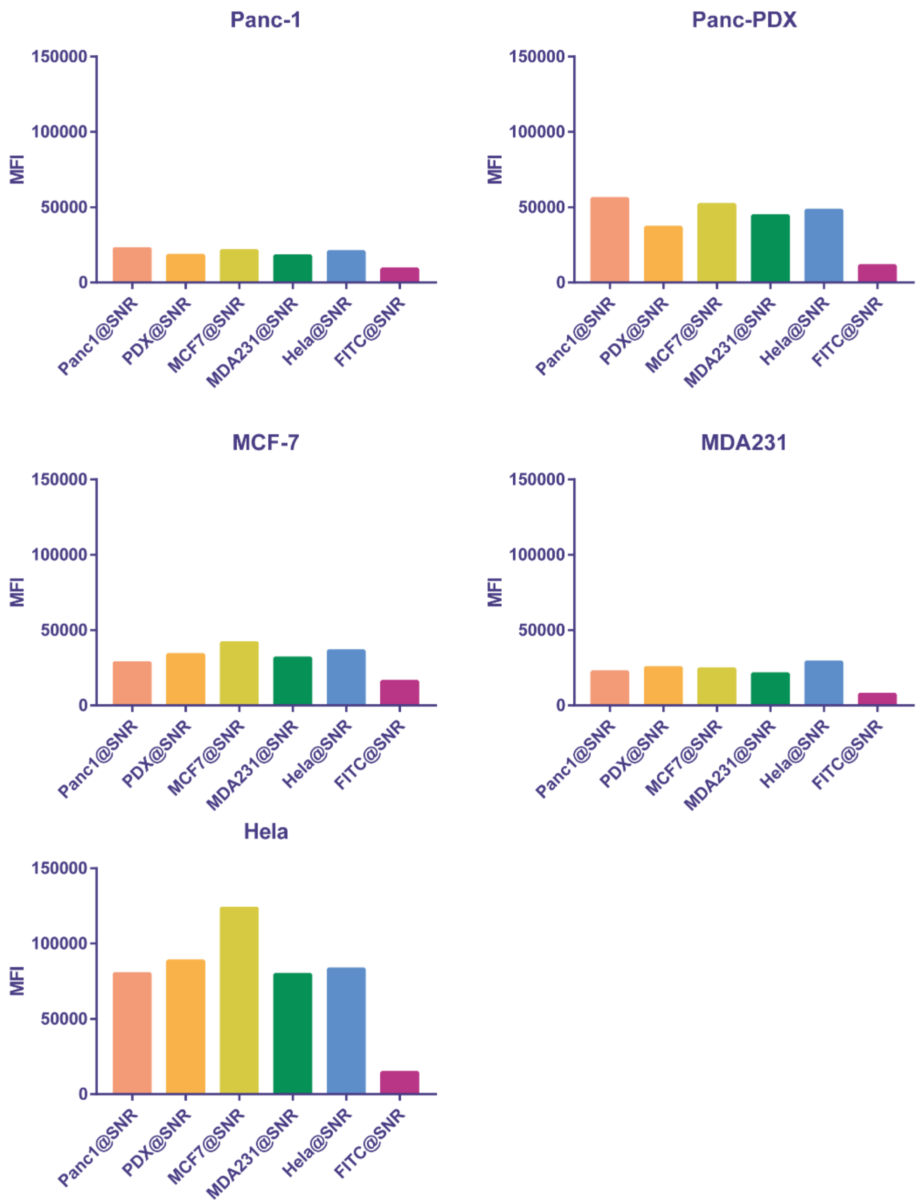

Figure S4C. FITC channel MFI of M-size type SiNRs internalized into the cells. The SiNR (FITC channel) mean of fluorescence intensity were calculated using FlowJo VX.

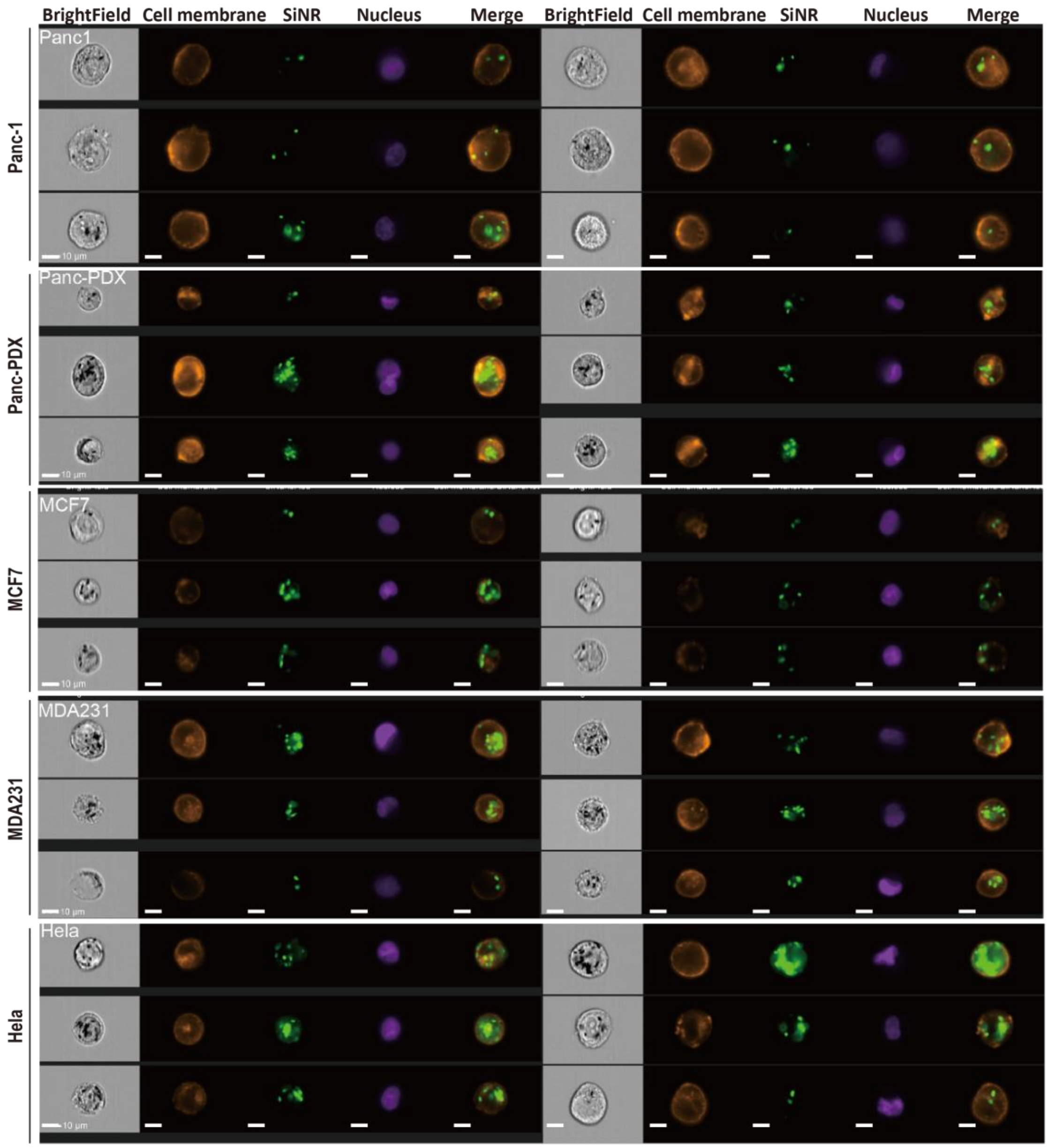

Figure S4D. Panc-PDX-PM-coating for M-size SiNR (green, FITC channel) localized within five cell types. Six selected imaging cytometry images were indicated per cell line. Hoechst (purple) indicates the cell nucleus as determined by cytometry. Alexa-594 (orange) conjugated WGA staining shows the cell membrane. Cell membrane/SiNR merged channel indicates the location of SiNR inside the cell. The panels in one group of images are as follows from left to right: bright field, cell membrane, SiNR, nucleus, and merge of cell membrane and SiNR. Scale bar represents 10 μm.

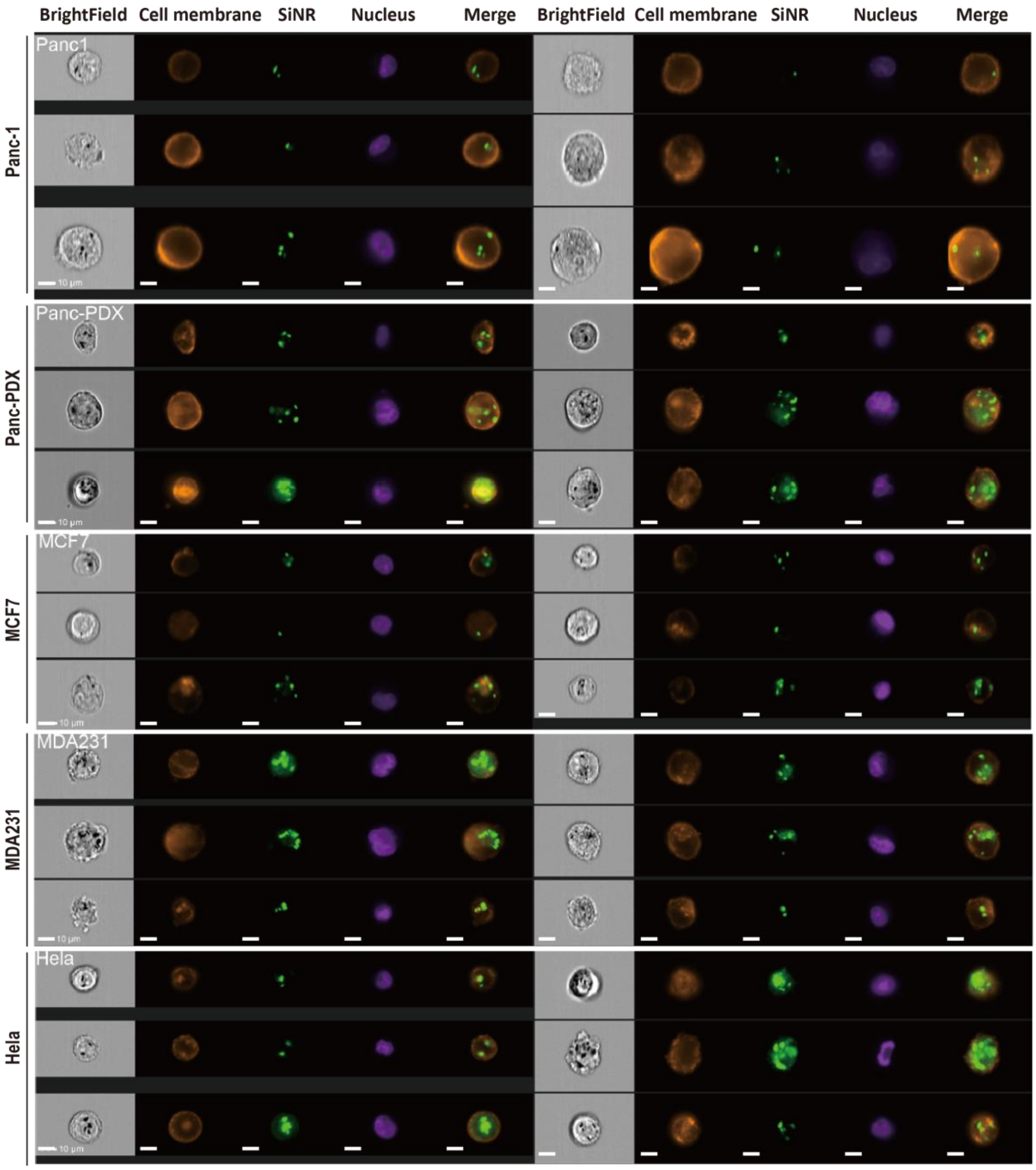

Figure S4E. MCF7-PM-coating for M-size SiNR (green, FITC channel) localized within five cell types. Six selected imaging cytometry images were indicated per cell line. Hoechst (purple) indicates the cell nucleus as determined by cytometry. Alexa-594 (orange) conjugated WGA staining shows the cell membrane. Cell membrane/SiNR merged channel indicates the location of SiNR inside the cell. The panels in one group of images are as follows from left to right: bright field, cell membrane, SiNR, nucleus, and merge of cell membrane and SiNR. Scale bar represents 10 μm.

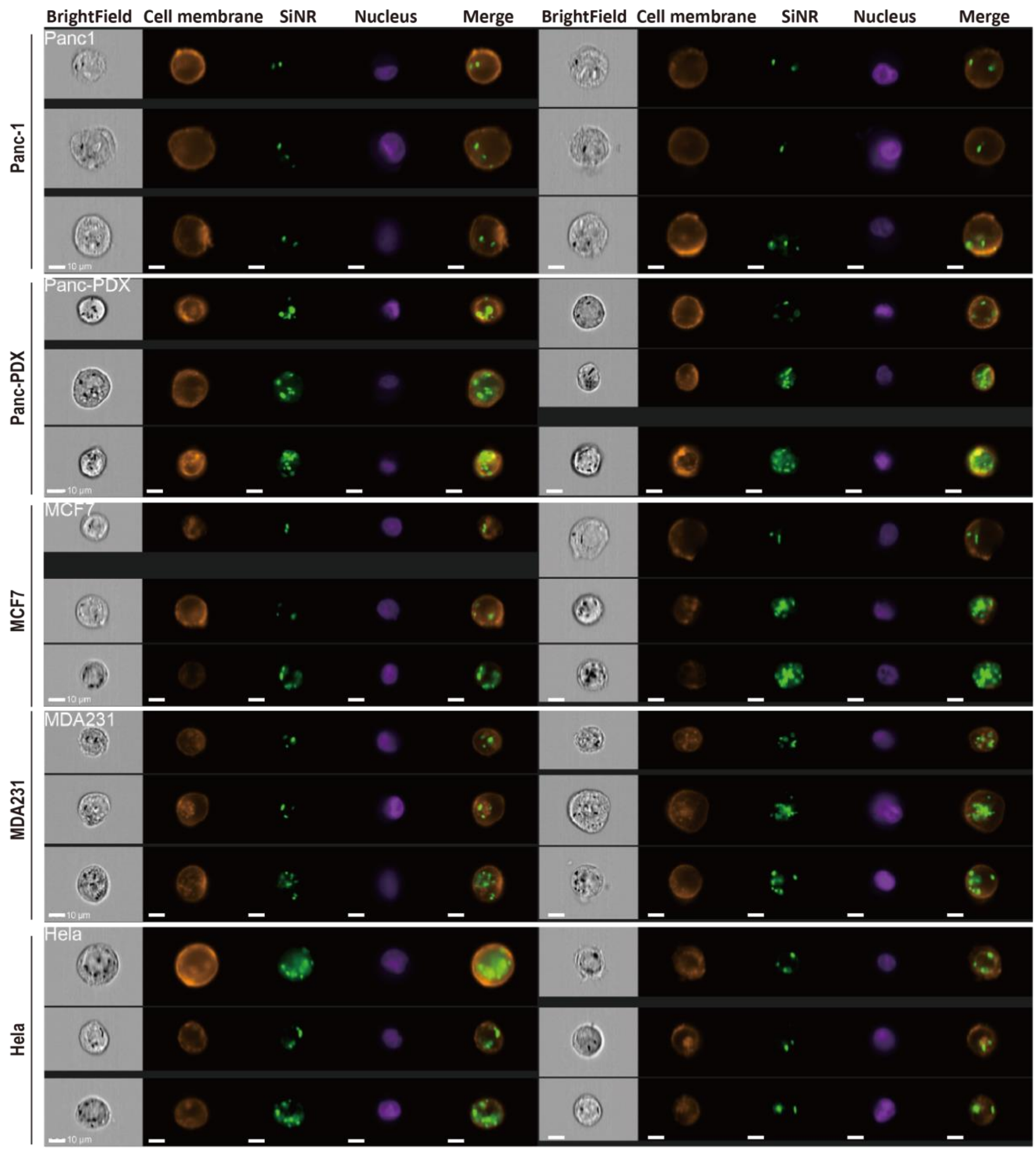

Figure S4F. MDA231-PM-coating for M-size SiNR (green, FITC channel) localized within five cell types. Six selected imaging cytometry images were indicated per cell line. Hoechst (purple) indicates the cell nucleus as determined by cytometry. Alexa-594 (orange) conjugated WGA staining shows the cell membrane. Cell membrane/SiNR merged channel indicates the location of SiNR inside the cell. The panels in one group of images are as follows from left to right: bright field, cell membrane, SiNR, nucleus, and merge of cell membrane and SiNR. Scale bar represents 10 μm.

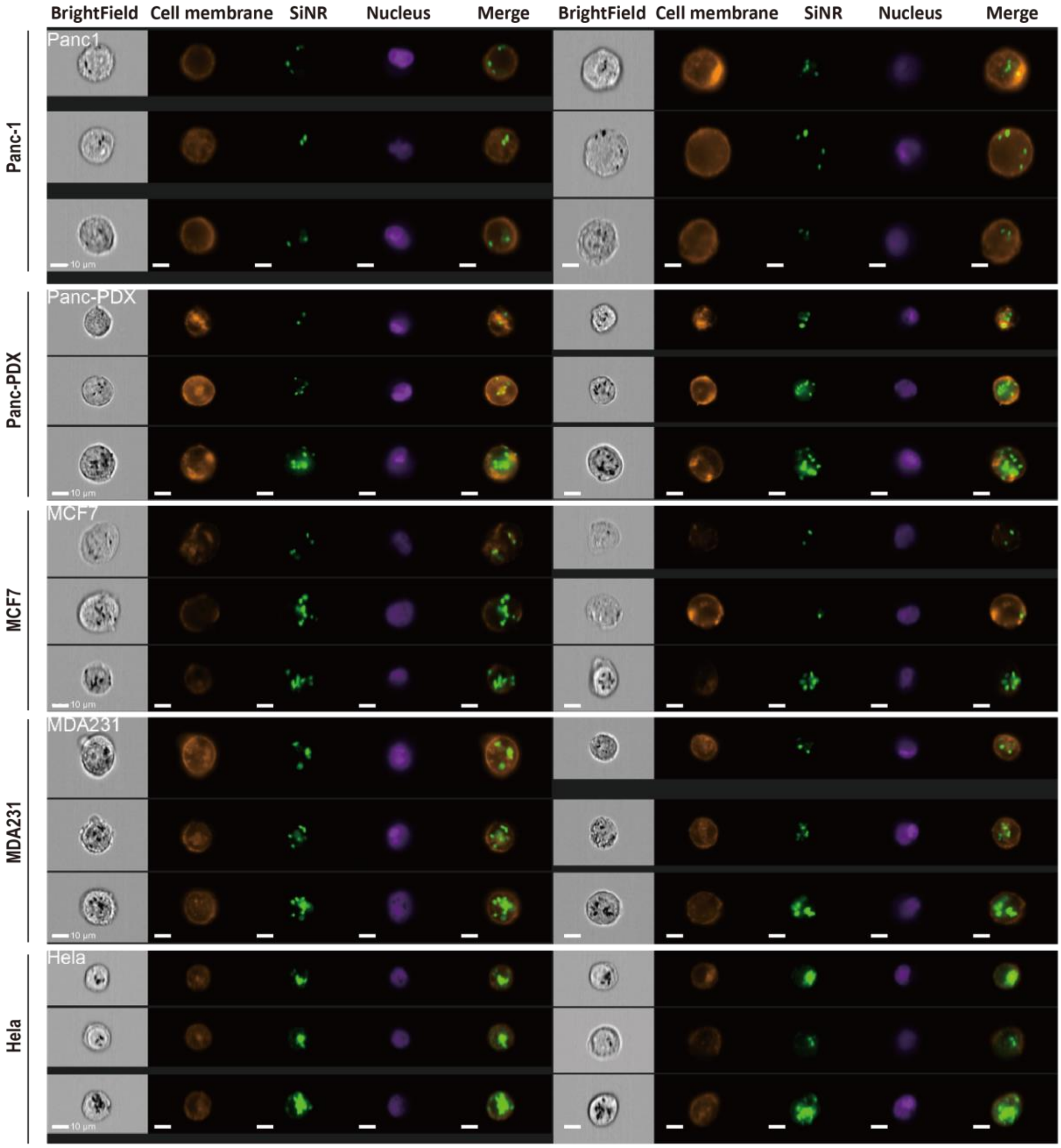

Figure S4G. Hela-PM-coating for M-size SiNR (green, FITC channel) localized within five cell types. Six selected imaging cytometry images were indicated per cell line. Hoechst (purple) indicates the cell nucleus as determined by cytometry. Alexa-594 (orange) conjugated WGA staining shows the cell membrane. Cell membrane/SiNR merged channel indicates the location of SiNR inside the cell. The panels in one group of images are as follows from left to right: bright field, cell membrane, SiNR, nucleus, and merge of cell membrane and SiNR. Scale bar represents 10 μm.

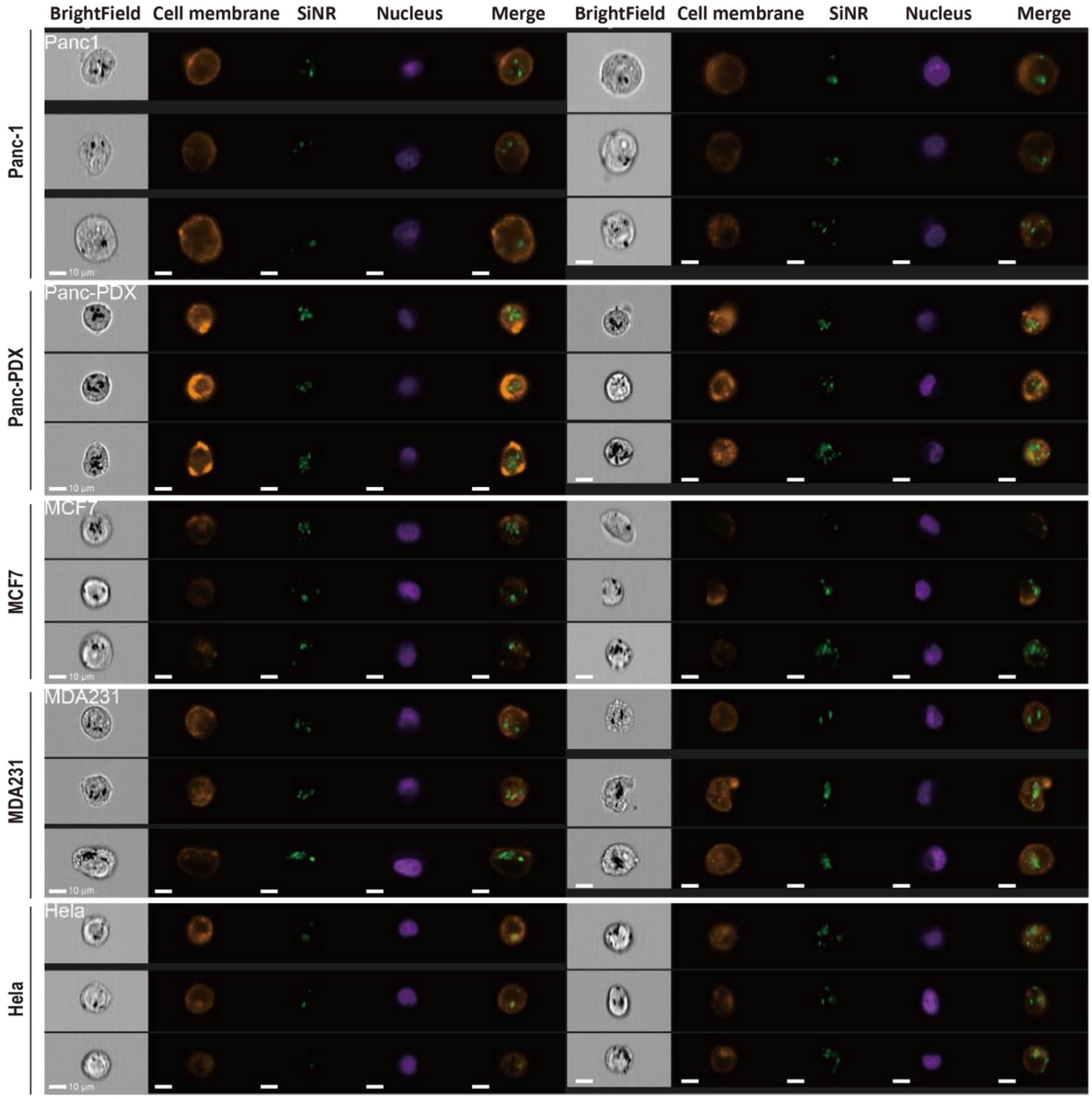

Figure S4H. Non-CMP-coating for M-size SiNR (green, FITC channel) localized within five cell types. Six selected imaging cytometry images were indicated per cell line. Hoechst (purple) indicates the cell nucleus as determined by cytometry. Alexa-594 (orange) conjugated WGA staining shows the cell membrane. Cell membrane/SiNR merged channel indicates the location of SiNR inside the cell. The panels in one group of images are as follows from left to right: bright field, cell membrane, SiNR, nucleus, and merge of cell membrane and SiNR. Scale bar represents 10 μm.

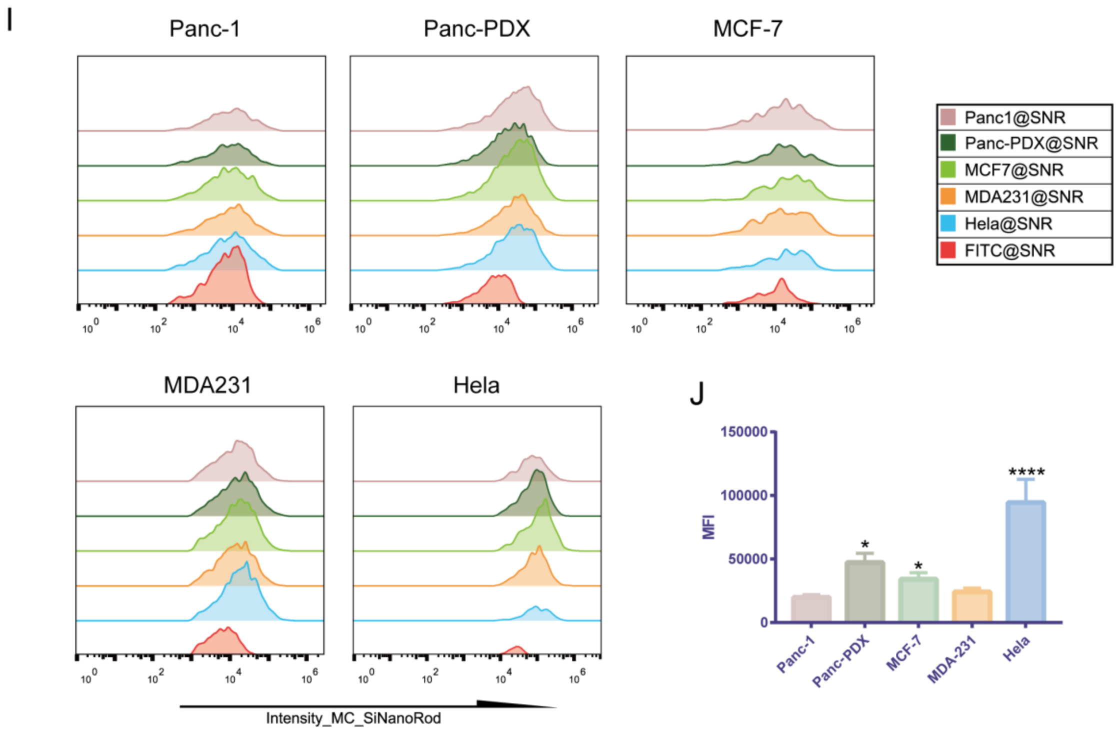

Figure S4I. (**I**) Histogram plot of M-size SiNR fluorescence intensity analysis. Raw data were exported from Amnis Ideas analysis software and analyzed in Flowjo VX. (**J**) Average MFI of M-size SiNR indicating internalization efficiency (*$p < 0.05$, ****$p < 0.0001$).

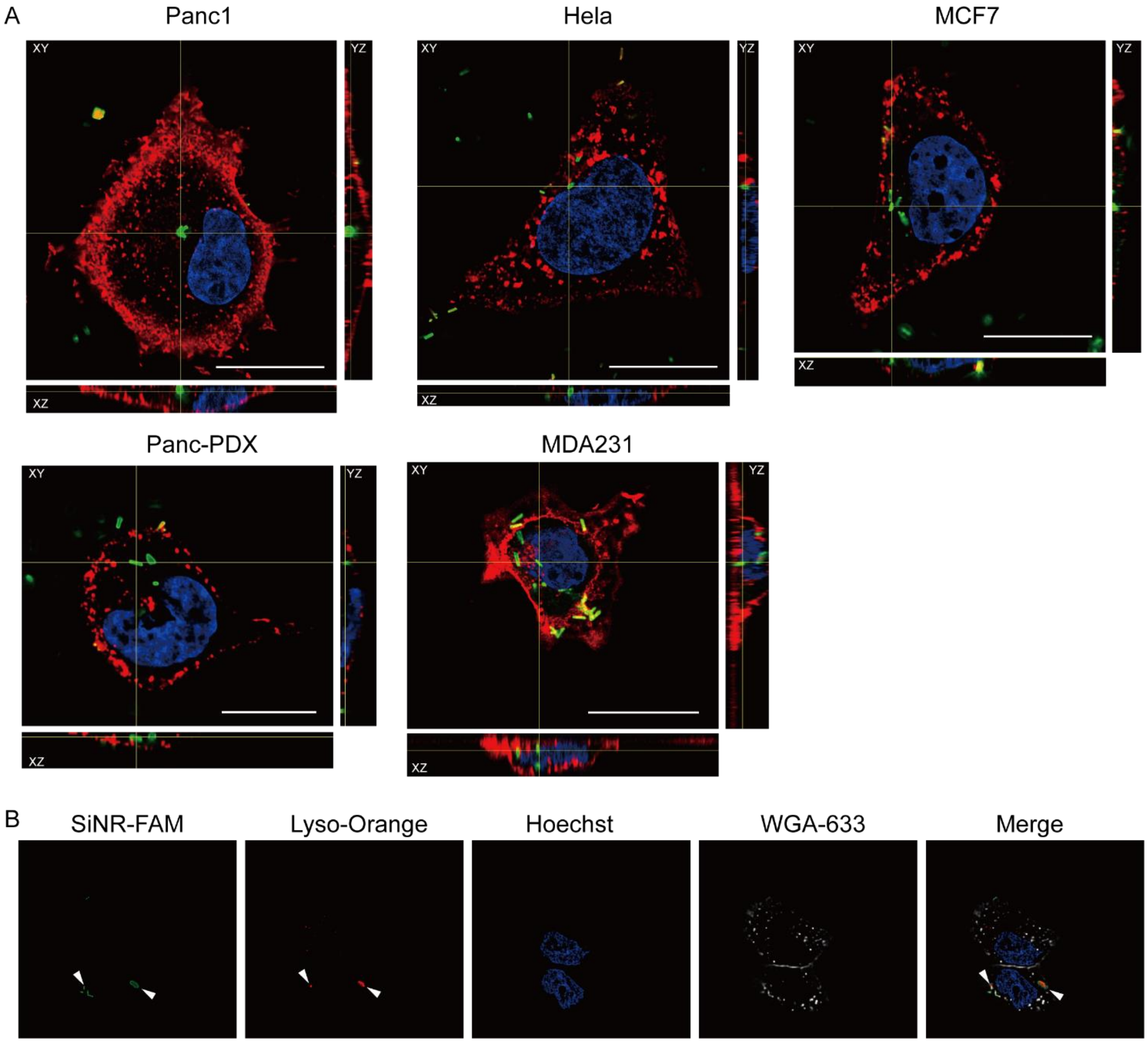

Figure S5. The detail confocal images of Panc1@SiNR internalized by five type cells. (**A**) Orthogonal views of five type cells to analyze the Panc1@SiNR uptake. Confocal Z-stack serial pictures were visualized as orthogonal section XY, YZ, and XZ planes. The crosshairs point the SiNR (in green) inside the cell body, observed in all three planes. The nuclei were labeled with Hoechst (in blue) and the cell membrane with Alexa-flour-633-conjucted WGA (in red). Scale bars correspond to 20 μm. (**B**) Colocalization of SiNR and lysosome in MCF7 cell. MCF7 cell were incubated with Panc1@SiNR (in green) for 6 h. Lysosome was stained with Lyso-Orange (in red), the nucleus were labeled with Hoechst (in blue), and the cell membrane with Alexa-flour-633-conjucted WGA (in white).

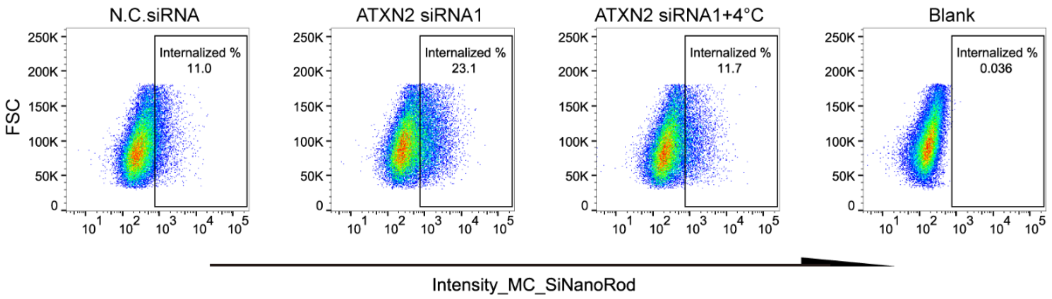

Figure S6. Spot plot of FSC (forward light scatter) vs. SiNR FAM fluorescence. Panc-1 cells with indicated treatment were incubated with Panc-PDX@SiNR. All cells subjected to analysis were first plotted by FSC vs. SSC (side light scatter), followed by gating for single cell population and then nucleus stained cells (Hoechst). "Internalized" gate was set for gating SiNR positive cells based on a blank control (no SiNR). For the 4 °C sample, cells were incubated with Panc-PDX@SiNR at 4 °C.

Table S1A. Stasis data of M-sized SiNR from imaging stream cytometry.

Treatment	Low% single cell	Medium% single cell	High% single cell	Internalized% single cell	Count All
Panc1-Panc1@SiNR	3.5062	7.2462	7.1439	17.9547	10000
Panc1-PancPDX@SiNR	3.8737	8.8567	7.6621	20.4437	10000
Panc1-MCF7@SiNR	3.0723	6.109	5.1678	14.4557	10000
Panc1-MDA231@SiNR	2.6739	6.1066	4.7696	13.5863	10000
Panc1-Hela@SiNR	3.0772	6.4944	5.3893	15.0969	10000
Panc1-FAM@SiNR	1.7369	2.7343	0.6363	5.1419	9523
PancPDX-Panc1@SiNR	40.6301	16.3921	4.8394	63.612	11849
PancPDX-PancPDX@SiNR	32.3478	21.3097	5.4534	60.0526	10185
PancPDX-MCF7@SiNR	36.5427	20.4728	7.2983	65.6844	12000
PancPDX-MDA231@SiNR	40.4728	20.0922	5.3618	67.3105	10000
PancPDX-Hela@SiNR	42.4975	17.8071	4.6091	66.3147	9942
PancPDX-FAM@SiNR	15.8722	12.3155	1.5485	29.9782	10000
MCF7-Panc1@SiNR	10.6977	12.2481	4.8062	27.7519	2003
MCF7-PancPDX@SiNR	11.0151	11.0151	6.9114	28.9417	2048
MCF7-MCF7@SiNR	17.8947	14.1053	4.8421	37.0526	2048
MCF7-MDA231@SiNR	13.0208	14.2361	6.0764	33.3333	2048
MCF7-Hela@SiNR	12.2396	14.0625	5.2083	31.5104	2048
MCF7-FAM@SiNR	3.25	5.5	1.75	10.5	2048
MDA231-Panc1@SiNR	7.6581	23.5043	14.2393	45.453	10000
MDA231-PancPDX@SiNR	11.9102	27.4163	12.7854	52.2831	10000
MDA231-MCF7@SiNR	11.2323	27.2017	15.0903	53.622	10000
MDA231-MDA231@SiNR	7.3425	25.6016	15.0212	48.0538	10000
MDA231-Hela@SiNR	13.3521	30.2801	14.8318	58.7458	10000
MDA231-FAM@SiNR	1.0895	10.5992	4.6226	16.3113	10000
Hela-Panc1@SiNR	52.6252	24.7863	4.0293	84.8596	4041
Hela-PancPDX@SiNR	64.1691	21.0682	2.5223	91.9881	4000
Hela-MCF7@SiNR	65.2112	17.5376	1.9327	91.0523	4000
Hela-MDA231@SiNR	61.2263	23.8052	2.1641	88.1876	4000
Hela-Hela@SiNR	65.7407	21.5278	0.9259	89.5833	1985
Hela-FAM@SiNR	10.3704	14.4444	1.8519	26.6667	2138

Table S1B. Stasis data of L-sized SiNRs from imaging stream cytometry.

Treatment	Low% single cell	Medium% single cell	High% single cell	Internalized% single cell	Count All
Panc1-Panc1@SiNR	9.2442	8.8663	3.9826	22.1802	7000
Panc1-PancPDX@SiNR	5.3047	7.4266	2.8868	15.6427	7000
Panc1-MCF7@SiNR	6.8593	7.7754	3.5001	18.1348	7000
Panc1-MDA231@SiNR	5.9912	5.8903	2.7264	14.6079	7000
Panc1-Hela@SiNR	6.6531	6.4246	2.7679	15.871	7000
Panc1-FAM@SiNR	0.2311	0.2311	0.0385	0.5008	7000
PancPDX-Panc1@SiNR	1.9952	12.4466	66.7933	84.6081	6000
PancPDX-PancPDX@SiNR	1.7255	13.9608	60.1569	77.8039	10000
PancPDX-MCF7@SiNR	2.5939	13.4471	62.3891	81.5017	6000
PancPDX-MDA231@SiNR	2.6968	12.0991	63.1924	82.1429	6000
PancPDX-Hela@SiNR	2.6777	11.6358	59.8832	78.1889	6000
PancPDX-FAM@SiNR	1.4184	4.154	1.8237	7.3961	6000
MCF7-Panc1@SiNR	4.9993	12.0571	13.5274	30.8337	10000
MCF7-PancPDX@SiNR	4.2034	11.2042	13.089	28.6313	10000
MCF7-MCF7@SiNR	4.772	11.9585	13.9611	30.8053	10000
MCF7-MDA231@SiNR	3.9293	10.5025	11.2328	25.7669	10000
MCF7-Hela@SiNR	4.48	10.5244	9.3156	24.4978	10000
MCF7-FAM@SiNR	1.0953	2.0537	1.0098	4.1759	10000
MDA231-Panc1@SiNR	7.1823	28.361	44.0147	80.0842	5000
MDA231-PancPDX@SiNR	12.6201	24.1512	15.6951	52.6372	8000
MDA231-MCF7@SiNR	10.9707	24.4439	17.998	53.64	8000
MDA231-MDA231@SiNR	10.9091	22.4324	19.1892	52.7273	8000
MDA231-Hela@SiNR	11.8067	23.9895	16.0368	51.9271	8091
MDA231-FAM@SiNR	1.2524	1.6378	0	2.8902	8000
Hela-Panc1@SiNR	5.0931	21.5553	39.4171	66.4695	10,000
Hela-PancPDX@SiNR	3.8329	18.174	31.5245	53.9047	10,000
Hela-MCF7@SiNR	7.138	23.1063	22.4512	52.7774	10,000
Hela-MDA231@SiNR	6.3974	22.501	26.458	55.6046	10,000
Hela-Hela@SiNR	5.1439	20.5076	35.3262	61.659	10,000
Hela-FAM@SiNR	0.2023	0.2832	0.0405	0.5259	10,000

Table S1B. Stasis data of S-sized SiNR from imaging stream cytometry.

Treatment	Low% single cell	Medium% single cell	High% single cell	Internalized% single	Count All
Panc1-Panc1@SiNR	1.8361	1.0112	0.3193	3.1666	7000
Panc1-PancPDX@SiNR	3.9519	3.3873	0.6627	8.002	7000
Panc1-MCF7@SiNR	2.7383	1.7308	0.3875	4.8566	7000
Panc1-MDA231@SiNR	2.8344	2.2611	0.5414	5.6369	7000
Panc1-Hela@SiNR	2.8137	2.5873	0.4851	5.9185	7000
Panc1-FAM@SiNR	1.7909	1.6464	0.2311	3.6684	7000
PancPDX-Panc1@SiNR	3.2258	17.1817	53.6503	75.8574	5000
PancPDX-PancPDX@SiNR	3.1976	16.6947	58.7681	80.1414	5000
PancPDX-MCF7@SiNR	3.6386	17.2836	53.2309	76.0038	5000
PancPDX-MDA231@SiNR	2.5024	13.0322	61.5535	79.948	5000
PancPDX-Hela@SiNR	1.7034	13.434	61.866	81.3008	5000
PancPDX-FAM@SiNR	1.8441	13.5003	60.9603	79.8191	5000
MCF7-Panc1@SiNR	2.2741	3.909	1.1742	7.3722	10,000
MCF7-PancPDX@SiNR	2.09	4.0631	1.5054	7.6586	10,000
MCF7-MCF7@SiNR	2.3306	3.7175	1.0438	7.1633	10,000
MCF7-MDA231@SiNR	1.2954	2.2484	0.4467	3.9905	10,000
MCF7-Hela@SiNR	2.129	3.2344	1.1873	6.5507	10,000
MCF7-FAM@SiNR	1.5478	4.4203	1.9646	7.9476	10,000
MDA231-Panc1@SiNR	3.4884	26.5592	50.6871	81.3425	6000
MDA231-PancPDX@SiNR	4.7871	26.138	35.9178	67.0191	6000
MDA231-MCF7@SiNR	4.8478	28.5841	40.3784	74.1354	6000
MDA231-MDA231@SiNR	6.686	25.8398	26.3566	59.0762	6000
MDA231-Hela@SiNR	4.8908	24.1464	38.4189	67.9176	6000
MDA231-FAM@SiNR	9.9127	26.7768	15.8042	52.6496	6000
Hela-Panc1@SiNR	4.811	18.666	10.1218	33.6301	10,000
Hela-PancPDX@SiNR	4.0496	13.4784	6.9961	24.524	10,000
Hela-MCF7@SiNR	4.8516	19.971	10.6155	35.496	10,000

Hela-MDA231@SiNR	3.5566	15.9968	7.7352	27.3046	10,000
Hela-Hela@SiNR	3.9178	14.1424	6.3864	24.4466	10,000
Hela-FAM@SiNR	3.3998	10.4045	2.7403	16.5445	10,000

Table S2. Common GO identifications shared in the five types of cells in gene enrichment.

Cell Type	GO ID	GO Term
	0044409	entry into host
	0001618	virus receptor activity
	0002474	antigen processing and presentation of peptide antigen via MHC class I
	0002478	antigen processing and presentation of exogenous peptide antigen
	0050839	cell adhesion molecule binding
	0098631	cell adhesion mediator activity
	0005924	cell-substrate adherens junction
	0005925	focal adhesion
	0023026	MHC class II protein complex binding
	0030055	cell-substrate junction
	0030100	regulation of endocytosis
	0030120	vesicle coat
	0030135	coated vesicle
Common	0030136	clathrin-coated vesicle
	0030662	coated vesicle membrane
	0030665	clathrin-coated vesicle membrane
	0030666	endocytic vesicle membrane
	0030670	phagocytic vesicle membrane
	0034329	cell junction assembly
	0034330	cell junction organization
	0042288	MHC class I protein binding
	0043112	receptor metabolic process
	0044291	cell-cell contact zone
	0045121	membrane raft
	0045296	cadherin binding
	0045335	phagocytic vesicle
	0072659	protein localization to plasma membrane

Table S3. Primers used in real-time PCR, hR18S and hTBP are reference genes.

Gene	Primer	Sequence
hATXN2	Forward	5'-AAGATATGGACTCCAGTTATGCAAA-3'
	Reverse	5'-CAAAGCCTCAAGTTCCTCAT-3'
hSAA1	Forward	5'-CTGCAGAAGTGATCAGCG-3'
	Reverse	5'-ATTGTGTACCCTCTCCCC-3'
hMELTF	Forward	5'-CCCTAAGTGGCGAGGACATTTAC-3'
	Reverse	5'-TCATCCAAGGTGAAGGCGTG-3'
hSRC	Forward	5'-ACCACCTTTGTGGCCCTCTATG-3'
	Reverse	5'-GCCACCAGTCTCCCTCTGTGTT-3'
hR18S	Forward	5'-AGAAACGGCTACCACATCCA-3'
	Reverse	5'-CACCAGACTTGCCCTCCA-3'
hTBP	Forward	5'-TGCACAGGAGCCAAGAGTGAA-3'
	Reverse	5'-CACATCACAGCTCCCCACCA-3'

Table S4. Small interfere RNA sequence targeting human ATXN2. The sequence of N.C. (negative control) is nontargeting in the human genome.

homo ATXN2		Sequence
siRNA1	Sense	5'-CCUCGAAAUCACAGAGUUUTT-3'
	Anti-sense	5'-AAACUCUGUGAUUUCGAGGTT-3'
siRNA2	Sense	5'-GCAUCGAACAGAGCUGUUATT-3'
	Anti-sense	5'-UAACAGCUCUGUUCGAUGCTT-3'
N.C.	Sense	5'-UUCUCCGAACGUGUCACGUTT-3'
	Anti-sense	5'-ACGUGACACGUUCGGAGAATT-3'
